# Simulating the impacts of interregional mobility restriction on the spatial spread of COVID-19 in Japan

**DOI:** 10.1038/s41598-021-97170-1

**Published:** 2021-09-23

**Authors:** Keisuke Kondo

**Affiliations:** Research Institute of Economy, Trade and Industry (RIETI), 1-3-1 Kasumigaseki, Chiyoda-ku, Tokyo, 100-8901 Japan

**Keywords:** Health policy, Epidemiology

## Abstract

A spatial susceptible–exposed–infectious–recovered (SEIR) model is developed to analyze the effects of restricting interregional mobility on the spatial spread of the coronavirus disease 2019 (COVID-19) infection in Japan. National and local governments have requested that residents refrain from traveling between prefectures during the state of emergency. However, the extent to which restricting interregional mobility prevents infection expansion is unclear. The spatial SEIR model describes the spatial spread pattern of COVID-19 infection when people commute or travel to a prefecture in the daytime and return to their residential prefecture at night. It is assumed that people are exposed to an infection risk during their daytime activities. The spatial spread of COVID-19 infection is simulated by integrating interregional mobility data. According to the simulation results, interregional mobility restrictions can prevent the geographical expansion of the infection. On the other hand, in urban prefectures with many infectious individuals, residents are exposed to higher infection risk when their interregional mobility is restricted. The simulation results also show that interregional mobility restrictions play a limited role in reducing the total number of infected individuals in Japan, suggesting that other non-pharmaceutical interventions should be implemented to reduce the epidemic size.

## Introduction

Non-pharmaceutical interventions (NPIs), which are public health measures for the prevention and control of infection, have played a key role in combating the coronavirus disease 2019 (COVID-19) pandemic^1–6^. To reduce the spatial spread of severe acute respiratory syndrome coronavirus 2 (SARS-CoV-2), national and local governments have enforced not only personal NPIs such as hand washing and mask-wearing, but also strict NPIs such as restrictions to movements, events, and travel, as well as school closures, and quarantine^7,8^. Among the strictest NPIs are lockdowns, which restrict social and economic activities and trigger severe economic downturns^3^.

National and local governments in Japan have requested that residents refrain from traveling between prefectures during the state of emergency to prevent the spread of COVID-19. The first state of emergency was declared on April 7, 2020 for seven prefectures (Saitama, Chiba, Tokyo, Kanagawa, Osaka, Hyogo, and Fukuoka), and it was extended to all 47 prefectures on April 16, 2020^8–10^. Although the restrictions on interregional mobility were relaxed after the first state of emergency was lifted in May 2020, the low level of lockdown restriction (i.e., voluntary compliance without penalties) was the first such experience in Japan since the Act on Special Measures for Pandemic Influenza and New Infectious Diseases Preparedness and Response was enacted after the A/H1N1 influenza pandemic in 2009^8,11,12^.

Furthermore, the second declaration of a state of emergency included the Greater Tokyo area (Saitama, Chiba, Tokyo, and Kanagawa) on January 7, 2021, and it was extended to the Greater Osaka area (Kyoto, Osaka, and Hyogo), Tochigi, Aichi, Gifu, and Fukuoka on January 13, 2021. The second state of emergency ended in Tochigi on February 7, 2021; in Gifu, Aichi, Kyoto, Osaka, Hyogo, and Fukuoka on February 28, 2021; and finally in the Greater Tokyo area on March 21, 2021. The third declaration came into effect on April 25, 2021 for Tokyo and the Greater Osaka area, and it was extended to Hokkaido, Aichi, Okayama, Hiroshima, Fukuoka, and Okinawa in May 2021. This state of emergency ended in nine of the aforementioned prefectures, with the exception of Okinawa, on June 20, 2021. However, a new declaration became effective, again, in Tokyo from July 12, 2021. As of July 27, 2021, Tokyo and Okinawa remain in a state of emergency (see Supplementary Appendix S1 for details). Although restrictions on interregional mobility between other prefectures have been relaxed while basic infection prevention measures are being maintained (e.g., keeping a safe distance between people, wearing masks, and hand washing), the national government has continued to request that residents avoid unnecessary travel between prefectures, especially to and from prefectures in a state of emergency^13^.

The present study aims to provide meaningful implications for combating the COVID-19 pandemic through interregional mobility restrictions. How interregional mobility restrictions limit the expansion of infection in Japan’s context is largely unknown, although many previous studies have emphasized the importance of understanding the spatial dynamics of spread^14–21^. Effective control measures that prevent spatial spread of SARS-CoV-2 are urgently demanded, and how NPIs such as travel restrictions and social distancing mitigate the epidemic must be investigated^22–31^.

To analyze how interregional mobility affects the spatial spread of SARS-CoV-2, we developed a spatial susceptible–exposed–infectious–recovered (SEIR) model. As the virus spreads through close contact from person to person (e.g., “breathing in air when close to an infected person who is exhaling small droplets and particles that contain the virus”, “having these small droplets and particles that contain virus land on the eyes, nose, or mouth, especially through splashes and sprays like a cough or sneeze”, and “touching eyes, nose, or mouth with hands that have the virus on them”)^32^, a spatial network of contagion was built by introducing interregional mobility into the standard SEIR model. The model assumes that people commute or travel to a region in the daytime and return to their residential region at night (e.g., people commute to a central business district on weekdays, whereas they travel to a rural resort area, visit a plaza in the suburbs for shopping and eating, or visit faraway relatives on holidays and weekends). Moreover, it assumes that people are exposed to the SARS-CoV-2 infection risk during their daytime activities, meaning that residents in one region are exposed to heterogeneous infection risks of SARS-CoV-2 depending on where they stay during the daytime.

To simplify the spatial network analysis, the interregional mobility is mathematically treated as an origin–destination (OD) matrix. It is demonstrated that the spatial SEIR model reduces to the standard SEIR model when the off-diagonal elements of the OD matrix are zero (i.e., when people remain in their residential regions). Therefore, the spatial SEIR model can be viewed as a generalized version of the standard SEIR model.

The approach developed can be also viewed as a spatial version of the social interaction approach. The social interaction approach constructs a contact matrix across various categories defined on the basis of factors such as age and gender^24,29,31,33–35^. Similarly, person-to-person interactions across different regions are incorporated into the OD matrix^22,27^.

The daily OD matrix is constructed from interregional mobility data obtained by geospatial information technology, namely, from the locational information of mobile phone users. The availability of mobility networks from mobile phone geolocation data advances the literature on infectious disease epidemiology^27,36,37^. These data capture the specific situations of individuals, such as commuting to work on weekdays and either remaining in the residential region or traveling to another region during the weekends. By tracking interregional mobility on each month, day, and time of day throughout one year prior to the pandemic, this study successfully captured the daily interregional mobility flows in the counterfactual situation.

This study aims to assess control measures based on a simulation analysis. Because mitigating the COVID-19 pandemic is an urgent priority, an epidemic model that guides the planning of efficient control measures is essential when few ideal data are available^6,35,38–40^. The spatial SEIR model in this study assumes that the past interregional mobility patterns will continue in future, regardless of how the COVID-19 pandemic evolves. By comparing findings simulated from the SEIR model under the free mobility assumption with those simulated under the strict interregional mobility restriction, this study evaluates how restricting movement mitigates the spatial spread of COVID-19 infection.

## Methods

### SEIR model without interregional mobility

In the absence of interregional mobility, the SEIR model assumes that infection in one region is independent of infection in all other regions. In other words, infection expansion in one region does not affect the infection dynamics in other regions. Later, this baseline model will be generalized to incorporate interregional mobility as a source of spatial infection expansion.

Suppose that there are $m$ regions and that region $i$ has population ${N}_{i}$. The total national population is expressed as $N={\sum }_{i=1}^{m}{N}_{i}$. The birth and death rates are excluded from the model, meaning that the national total population is fixed over time. The regional population distribution is also fixed because residential change is forbidden in this model. Daily mobility (e.g., commuting and travel) is similarly forbidden in the baseline SEIR model, but this assumption will be relaxed later.

Let ${S}_{i}\left(t\right),$ ${I}_{i}(t)$, and ${R}_{i}(t)$ denote the number of susceptible, infectious, and recovered residents, respectively, in region $i$ at date $t$. Let ${E}_{i}(t)$ denote the number of residents in region $i$ at date $t$ who have been exposed to the COVID-19 infection, but who are in the latent period and not yet infectious. In the SEIR model without a spatial network, the epidemic dynamics are expressed as follows:

1 $$\begin{aligned}&\frac{{\mathrm{d}}{S}_{i}\left(t\right)}{{\mathrm{d}}t}=-\beta \frac{{S}_{i}\left(t\right){I}_{i}\left(t\right)}{{N}_{i}},\\ &\frac{{\mathrm{d}}{E}_{i}\left(t\right)}{{\mathrm{d}}t}=\beta \frac{{S}_{i}\left(t\right){I}_{i}\left(t\right)}{{N}_{i}}-\varepsilon {E}_{i}\left(t\right),\\ &\frac{{\mathrm{d}}{I}_{i}\left(t\right)}{{\mathrm{d}}t}=\varepsilon {E}_{i}\left(t\right)-\gamma {I}_{i}\left(t\right),\\ &\frac{{\mathrm{d}}{R}_{i}\left(t\right)}{{\mathrm{d}}t}=\gamma {I}_{i}\left(t\right),\\&{S}_{i}\left(t\right)+{E}_{i}\left(t\right)+{I}_{i}\left(t\right)+{R}_{i}\left(t\right)={N}_{i},\\ &\sum _{k=1}^{m}{N}_{k}=N,\end{aligned}$$

where $\beta$ is the transmission rate parameter, $\varepsilon$ is the incubation rate, and $\gamma$ is the recovery rate.

The SEIR model without interregional mobility gives the baseline results for comparison with the extended model described below. A main distinguishing feature of the spatial SEIR model is the force of infection $\lambda (t)$, which measures the rate at which susceptible individuals contract the infection. This term in the standard SEIR model, namely $\beta {I}_{i}(t)/{N}_{i}$ is independent of infection in all other regions. We highlight how allowing for interregional mobility changes the specified force of infection.

### SEIR model with interregional mobility

This study introduces interregional mobility as a spatial network of contagion into the standard SEIR model. The basic assumptions are those of the above-mentioned baseline setting. As an additional assumption, interregional mobility causes geographical infection expansion through person-to-person contact.

Individuals are assumed to be exposed to infection risk only in the region they occupy in the daytime. For example, suppose that an individual lives in one region and commutes to another region during the daytime. This interregional mobility provides two possible transmission channels: transfer of the disease from the region occupied by the commuting individual to the residential region and spread of the disease from the residential region to the region occupied by the commuting individual. Thus, interregional mobility spreads the infection disease across spatial domains.

Interregional mobility can be modeled by spatial network analysis. Let ${{\varvec{\pi}}}^{h}(t)$ denote the OD probability matrix across regions on date $t$:

2 $${{\varvec{\pi}}}^{h}\left(t\right)=\left(\begin{array}{cccc}{\pi }_{\mathrm{1,1}}^{h}\left(t\right)& {\pi }_{\mathrm{1,2}}^{h}\left(t\right)& \cdots & {\pi }_{1m}^{h}\left(t\right)\\ {\pi }_{\mathrm{2,1}}^{h}\left(t\right)& {\pi }_{\mathrm{2,2}}^{h}\left(t\right)& \cdots & {\pi }_{2m}^{h}\left(t\right)\\ \vdots & \vdots & \ddots & \vdots \\ {\pi }_{m1}^{h}\left(t\right)& {\pi }_{m2}^{h}\left(t\right)& \cdots & {\pi }_{mm}^{h}\left(t\right)\end{array}\right),$$

where ${\pi }_{ij}^{h}(t)$ is the probability of traveling from region $i$ to region $j$ on date $t$ for an individual with state $h \in \{S, E, I, R\}$. The elements of ${{\varvec{\pi}}}^{h}\left(t\right)$ along a row must sum to one by the definition of probability. To simplify the calculation, we assume that this probability matrix is independent of the infection conditions over time (i.e., is not endogenous). However, the model admits exogenous seasonal variations or NPIs such as interregional mobility restrictions.

In terms of the OD matrix, the expected mobility flow from region $i$ to region $j$ and the expected daytime population in each region can be calculated. First, the mobility flows of susceptible, exposed, infectious, and recovered individuals from region $i$ to region $j$ can be calculated as follows, respectively:

3 $$\begin{aligned}&{S}_{ij}\left(t\right)={{\pi }_{ij}^{S}\left(t\right)S}_{i}(t),\\ &{E}_{ij}\left(t\right)={\pi }_{ij}^{E}\left(t\right){E}_{i}\left(t\right),\\ &{I}_{ij}\left(t\right)= {\pi }_{ij}^{I}\left(t\right){I}_{i}\left(t\right),\\ &{R}_{ij}\left(t\right)= {\pi }_{ij}^{R}\left(t\right){R}_{i}\left(t\right).\end{aligned}$$

Each expected number in region $i$ in the daytime is calculated as follows:

4 $$\begin{aligned}&{\tilde{S }}_{i}\left(t\right)=\sum_{k=1}^{m}{S}_{ki}\left(t\right),\\ &{\tilde{E }}_{i}\left(t\right)=\sum_{k=1}^{m}{E}_{ki}\left(t\right),\\ &{\tilde{I }}_{i}\left(t\right)= \sum_{k=1}^{m}{I}_{ki}(t),\\ &{\tilde{R }}_{i}\left(t\right)= \sum_{k=1}^{m}{R}_{ki}\left(t\right).\end{aligned}$$

The expected population in region $i$ in the daytime is calculated as follows:

5 $${\tilde{N }}_{i}\left(t\right)={\tilde{S }}_{i}\left(t\right)+{\tilde{E }}_{i}\left(t\right)+{\tilde{I }}_{i}\left(t\right)+{\tilde{R }}_{i}\left(t\right).$$

The OD probability matrix ${\pi }_{ij}^{h}(t)$ is simply assumed to be the same among susceptible, exposed, infectious, and recovered individuals in the baseline simulation. In the extended simulation, we assume that the government would restrict the interregional mobility of infectious individuals to remain in their residential communities. In other words, the OD probability matrix ${\pi }_{ij}^{I}(t)$ becomes the identity matrix. This assumption is reasonable because the government requests patients with cold-like symptoms to refrain from going to the office or traveling. Note that the use of the identity matrix only restricts interregional mobility and does not mean isolation and quarantine. It is assumed that infectious individuals would continue their daytime activities within their residential regions.

Importantly, the spatial distribution of infectious individuals in the daytime affects the infection risk in each region. By nighttime, infectious individuals have returned to their residential regions. Therefore, we count cases of the infection within the residential regions. When residents in region $i$ are exposed to the heterogeneous infection risk in each region that they occupy during the daytime, the infection dynamics in region $i$ are given by

6 $$\begin{aligned}&\frac{{\mathrm{d}}{S}_{i}\left(t\right)}{\mathrm{d}t}=\sum_{k=1}^{m}\frac{\mathrm{d}{S}_{ik}\left(t\right)}{\mathrm{d}t} ,\end{aligned}$$

where $\mathrm{d}{S}_{ij}\left(t\right)/\mathrm{d}t$ represents the transition that susceptible individuals residing in region $i$ and staying in region $j$ during the daytime become infected, and the sum of them in terms of region $j$ represents the transition in residential region $i$.

Consider residents in region $i$ who remain in region $i$. The infection dynamics are expressed as follows:

7 $$\begin{aligned}&\frac{{\mathrm{d}}{S}_{ii}\left(t\right)}{\mathrm{d}t}=-\beta \frac{{\tilde{I }}_{i}\left(t\right)}{{\tilde{N }}_{i}\left(t\right)}{S}_{ii}\left(t\right),\end{aligned}$$

where the force of infection $\beta {\tilde{I }}_{i}\left(t\right)/{\tilde{N }}_{i}\left(t\right)$ depends on the number of infectious individuals and population in region $i$ in the daytime. The infection dynamics for individuals who reside in region $i$ and stay in region $j$ in the daytime are expressed as

8 $$\begin{aligned}&\frac{\mathrm{d}{S}_{ij}\left(t\right)}{\mathrm{d}t}=-\beta \frac{{\tilde{I }}_{j}\left(t\right)}{{\tilde{N }}_{j}\left(t\right)}{S}_{ij}\left(t\right),\end{aligned}$$

where the force of infection $\beta {\tilde{I }}_{j}\left(t\right)/{\tilde{N }}_{j}\left(t\right)$ depends on the number of infectious individuals and population in region $j$ in the daytime. Thus, residents in region $i$ are exposed to heterogeneous infection risks.

The overall transmission of infection in region $i$ is expressed as the sum of the transmission of infection in terms of each outflow, which is given by

9 $$\begin{aligned}&\frac{\mathrm{d}{S}_{i}\left(t\right)}{\mathrm{d}t}=-\beta \sum_{k=1}^{m}\frac{{\tilde{I }}_{k}\left(t\right)}{{\tilde{N }}_{k}\left(t\right)}{S}_{ik}\left(t\right).\end{aligned}$$

Finally, the dynamic system of equations of the spatial SEIR model with interregional mobility is given by

10 $$\begin{aligned} & \frac{\mathrm{d}{S}_{i}\left(t\right)}{\mathrm{d}t}=-\beta \sum_{k=1}^{m}\frac{{\tilde{I }}_{k}\left(t\right)}{{\tilde{N }}_{k}\left(t\right)}{S}_{ik}(t),\\ &\frac{\mathrm{d}{E}_{i}\left(t\right)}{\mathrm{d}t}=\beta \sum_{k=1}^{m}\frac{{\tilde{I }}_{k}\left(t\right)}{{\tilde{N }}_{k}\left(t\right)}{S}_{ik}(t)-\varepsilon {E}_{i}\left(t\right),\\ &\frac{\mathrm{d}{I}_{i}\left(t\right)}{\mathrm{d}t}=\varepsilon {E}_{i}\left(t\right)-\gamma {I}_{i}\left(t\right),\\ &\frac{\mathrm{d}{R}_{i}\left(t\right)}{\mathrm{d}t}=\gamma {I}_{i}\left(t\right),\\ &{S}_{i}\left(t\right)+{E}_{i}\left(t\right)+{I}_{i}\left(t\right)+{R}_{i}\left(t\right)={N}_{i},\\ &\sum _{k=1}^{m}{N}_{k}=N.\end{aligned}$$

When interregional mobility is restricted, the diagonal and off-diagonal elements of the OD matrix ${{\varvec{\pi}}}^{h}\left(t\right)$ take values 1 and 0, respectively. Under this assumption, we see that ${N}_{ij}\left(t\right)={S}_{ij}\left(t\right)={E}_{ij}\left(t\right)={I}_{ij}\left(t\right)={R}_{ij}\left(t\right)=0$ for all $j(\ne i)$ and that ${\tilde{N }}_{i}\left(t\right)={N}_{i}$, ${\tilde{S }}_{i}\left(t\right)={S}_{i}\left(t\right)$, ${\tilde{E }}_{i}\left(t\right)={E}_{i}\left(t\right)$, ${I}_{i}\left(t\right)={I}_{i}\left(t\right)$, and ${\tilde{R }}_{i}\left(t\right)={R}_{i}\left(t\right)$. That is, the spatial SEIR model with interregional mobility reduces to the baseline SEIR model. Thus, the spatial SEIR model with interregional mobility can thus be viewed as a generalized version of the SEIR model.

### Simulation setting

The study objective was to evaluate how interregional mobility restrictions arrest the spatial spread of COVID-19 infection. For this purpose, we compared the simulation results of the spatial SEIR model with and without interregional mobility. The difference between the two sets of results revealed the impact of interregional mobility on the spread of COVID-19 infection.

To uncover the condition under which restricting the interregional mobility effectively prevents spatial infection spread, we also considered another type of NPI implemented. The effectiveness of the NPIs (scaling factor of the transmission rate) is defined as $\alpha \left(t\right)$, and the time-varying transmission rate is given by $\beta \left(t\right)=\alpha \left(t\right)\beta$. Note that we do not consider heterogeneous effectiveness of the NPIs in each region in this study, because we focus only on how heterogeneous contact rates resulting from interregional mobility affect the spatial infection expansion.

The parameters settings of the simulation analysis are as follows. Average incubation and infectious periods ${\ell}_{\varepsilon }$ and ${\ell}_{\gamma }$ were assumed as 5 days and 10 days, respectively^3,39,41–43^. The infectiousness probability of an exposed individual was given as $\varepsilon =1/{\ell}_{\varepsilon }$, and the recovery probability of an infected individual was given by $\gamma =1/{\ell}_{\gamma }$. The transmission rate was determined as $\beta ={\mathcal{R}}_{0}\gamma$ based on the standard SEIR model. The basic reproduction number ${\mathcal{R}}_{0}$ was set to 2.6^41,44^, close to that obtained by other studies in Japan^45,46^. These parameter settings were common to all simulation scenarios.

In this study, the effectiveness of NPIs is given exogenously by the parameter, $\alpha \left(t\right)$, which can be viewed as a future policy target parameter and a parameter that captures the cyclical trend observed in the previous year. To guide more effective policy responses to COVID-19, this study assumes that the seasonal trend of infection and NPIs observed in 2020 and 2021 will continue until December 31, 2023. Note that the impact of vaccination and the appearance of new variants were not considered in this long-run simulation.

This study estimated the parameter $\alpha \left(t\right)$ from the effective reproduction number ${\mathcal{R}}_{e}(t)$. The effective reproduction number reflects the effectiveness of NPIs because ${\mathcal{R}}_{e}(t)=\alpha (t){\mathcal{R}}_{0}$^41,47^. For example, when declaring a state of emergency on April 7, 2020, the government requested that person-to-person contact be reduced by at least 70%, meaning that the effectiveness of intervention corresponded to $\alpha \left(t\right)=0.3$. Therefore, this study takes the ratio of the effective and basic reproduction numbers to estimate the daily values of the parameter $\alpha \left(t\right)$.

The effective reproduction number was taken from open data offered by the Toyo Keizai Inc^48^. Their estimation method was based on the dispersibility ratio^49^. To mitigate the extreme fluctuations in the effective reproduction number during the early stages of the COVID-19 outbreak in rural prefectures, this study focused on the effective reproduction number in Tokyo between June 1, 2020 and May 31, 2021. The monthly average was calculated and inserted into each day of the corresponding month in the simulation period. A final process was to calculate the rolling average with a window time of 21 days to smooth the discontinuity between months.

**Figure 1:**
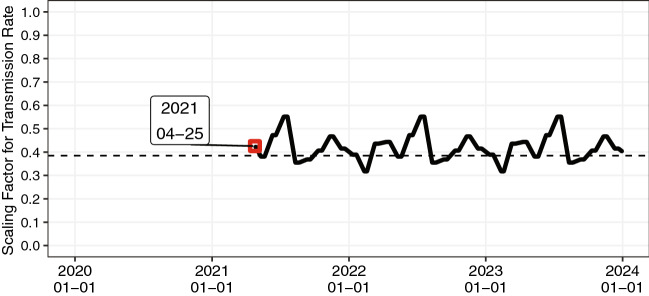
Simulation settings of the effectiveness of the non-pharmaceutical interventions. The simulations were started on April 25, 2021. The dashed line $1/{\mathcal{R}}_{0}$ corresponds to an effective reproduction number with the value of one. The effective reproduction number in Tokyo, which was calculated and provided by the Toyo Keizai Inc.^48^, was used to estimate the effectiveness of the NPIs.

Figure 1 presents the effectiveness of NPIs, the scaling factor of the transmission rate, estimated from the effective reproduction number. The simulation was started on April 25, 2021, which is the day of the third declaration of a state of emergency in Tokyo, Kyoto, Osaka, and Hyogo. Given the basic reproduction number, ${\mathcal{R}}_{0}$, the inverse ${1/\mathcal{R}}_{0}$ becomes a threshold for whether the number of new infections increases or decreases (i.e., whether the effective reproduction number is less than or greater than one). However, Fig. 1 shows that the scaling factor is greater than one in most months, suggesting that the infection will continue to expand in the long run.

### Data

Positive COVID-19 cases in each prefecture of Japan are reported by the prefectural governments and the Ministry of Health, Labour and Welfare (MHLW). This study used daily and cumulative numbers of positive tests collected by the NHK (Japan Broadcasting Corporation)^50^. The data was downloaded on June 28, 2021. Slight differences in the reported numbers were observed between the prefectural governments, the MHLW, and the NHK, owing to the timing of counting. However, they did not affect the qualitative results. It is also essential that the reported positive cases are connected to the residential prefectures to maintain consistency with the theoretical assumptions, but a small part of them do not necessarily coincide with the residential locations because some travelers were tested for COVID-19 in different prefectures during the holiday season.

**Figure 2:**
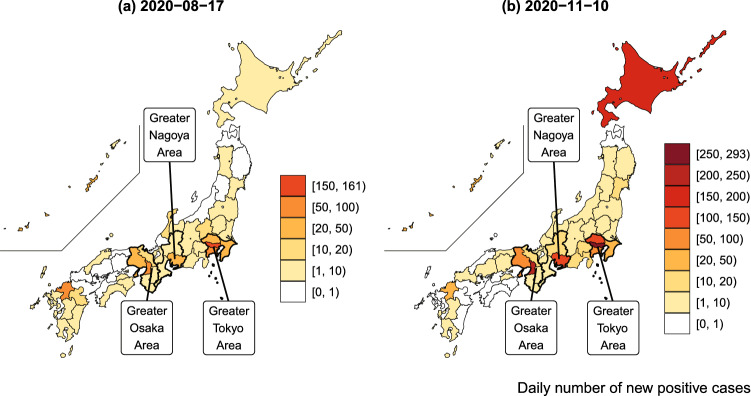
COVID-19 infections across prefectures. This map was constructed by the author from data of the daily number of positive tests. The original data on COVID-19 cases was compiled from the NHK^50^.

Figure 2 is a snapshot of the geographical distribution of the numbers of positive cases on August 17, 2020 and November 10, 2020. The COVID-19 infection numbers were highest in Tokyo (161 and 293 patients testing positive, respectively), followed by Osaka. Although the infection tended to expand in prefectures with large cities, such as Tokyo, Osaka, Nagoya, Sapporo, Yokohama, and Saitama, other prefectures occasionally experienced a sudden rise in infection numbers.

Considering the observed cumulative number of positive patients and the average number of days in the incubation and infectious periods $({\ell}_{\varepsilon }$ and ${\ell}_{\gamma }$, respectively), the variables in the SEIR model were constructed as follows:

11 $$\begin{aligned} &{R}_{i}\left(t\right)={P}_{i}\left(t-{\ell}_{\gamma }\right),\\ &{I}_{i}\left(t\right)={P}_{i}\left(t\right)-{R}_{i}\left(t\right),\\ &{E}_{i}\left(t\right)={P}_{i}\left(t+{\ell}_{\varepsilon }\right)-{P}_{i}\left(t\right),\\ &{S}_{i}\left(t\right)={N}_{i}-{E}_{i}\left(t\right)-{I}_{i}\left(t\right)-{R}_{i}\left(t\right)\end{aligned}$$

where ${P}_{i}(t)$ represents the cumulative number of positive tests reported in prefecture $i$ until date $t$. The total population in each prefecture $i$ was based on data from October 2019 and was fixed over time (i.e., no migration was assumed across prefectures). These variables were calculated from the observed data before the start date of the simulation.

The theoretical part of the model was based on the probabilistic mobility of individuals, but the OD probability matrix in the empirical part was estimated from observed data on interregional mobility, which were derived from the locational information of mobile phone users. The Regional Economy and Society Analyzing System (RESAS), a web application developed by the Headquarters for Overcoming Population Decline and Vitalizing Local Economy in Japan at the Prime Minister's Office, was released on April 21, 2015^51^. The RESAS app visualizes many types of data in Japan, including a dynamic map of inter-municipal human flows (the From–To Analysis) based on the Mobile Spatial Statistics of NTT DOCOMO^52^. The detailed information of inter-municipal flows are available by gender, age, year, month, day of the week (weekdays and weekends), and time of day (4 am, 10 am, 2 pm, 8 pm).

This study applied the monthly data of interregional flows from September 2015 through August 2016 by day of week (weekday or weekend). Although the interregional flow data up to January 2021 is visualized on the RESAS app as of June 2021, the application programming interface can download the original data only within a restricted period (from September 2015 to August 2016). The daytime population in each prefecture was estimated from the inter-municipal flows observed at 2 pm. The inter-municipal flows were aggregated into inter-prefectural flows to match the observational unit of the COVID-19 infection data. In each scenario, the same daily pattern of interregional mobility from September 2015 to August 2016 was assumed from the start date of the simulation through December 31, 2023; that is, the same mobility pattern was repeated on the same day of each year.

From the data, we calculated the share of inter-prefectural mobility that matched the probability of interregional mobility. Let ${\varvec{C}}(t)$ denote the OD matrix across the 47 prefectures on date $t$:

12 $${\varvec{C}}\left(t\right)=\left(\begin{array}{cccc}{c}_{\mathrm{1,1}}\left(t\right)& {c}_{\mathrm{1,2}}\left(t\right)& \cdots & {c}_{\mathrm{1,47}}\left(t\right)\\ {c}_{\mathrm{2,1}}\left(t\right)& {c}_{\mathrm{2,2}}\left(t\right)& \cdots & {c}_{\mathrm{2,47}}\left(t\right)\\ \vdots & \vdots & \ddots & \vdots \\ {c}_{47,1}\left(t\right)& {c}_{47,2}\left(t\right)& \cdots & {c}_{47,47}\left(t\right)\end{array}\right),$$

where ${c}_{ij}(t)$ represents the mobility flow (i.e., number of people) from prefecture $i$ to prefecture $j$ on date $t$. This OD matrix was row-standardized to express the share as follows:

13 $${\varvec{W}}\left(t\right)=\left(\begin{array}{cccc}{w}_{\mathrm{1,1}}\left(t\right)& {w}_{\mathrm{1,2}}\left(t\right)& \cdots & {w}_{1,47}\left(t\right)\\ {w}_{\mathrm{2,1}}\left(t\right)& {w}_{\mathrm{2,2}}\left(t\right)& \cdots & {w}_{2,47}\left(t\right)\\ \vdots & \vdots & \ddots & \vdots \\ {w}_{47,1}\left(t\right)& {w}_{47,2}\left(t\right)& \cdots & {w}_{\mathrm{47,47}}\left(t\right)\end{array}\right),$$

where ${w}_{ij}\left(t\right)$ represents the share of residents in prefecture $i$ who were staying in prefecture $j$ on date $t$. The row-standardization of ${\varvec{C}}(t)$ gives the share of inter-prefectural flows as follows:

14 $${w}_{ij}\left(t\right)=\frac{{c}_{ij}\left(t\right)}{\sum_{k=1}^{47}{c}_{ik}\left(t\right) }.$$

This weight matrix corresponds to the probabilistic OD matrix in the theoretical model. This study considered ${{\varvec{\pi}}}^{S}\left(t\right)={{\varvec{\pi}}}^{E}\left(t\right)={{\varvec{\pi}}}^{I}\left(t\right)={{\varvec{\pi}}}^{R}\left(t\right)=\boldsymbol{ }{\varvec{W}}\left(t\right)$ in the baseline simulation. However, this assumption was modified in the extended simulation analysis. In this extension, the OD matrix becomes the identity matrix only for infectious persons, meaning that infectious individuals are restricted to their residential prefectures.

**Figure 3:**
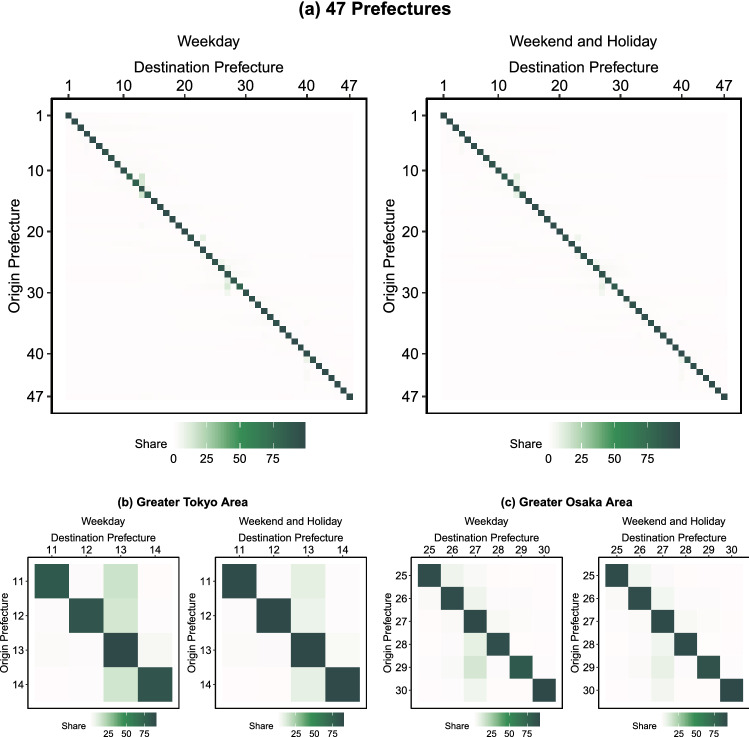
Origin–Destination matrix between prefectures, constructed from the interregional mobility data obtained by the “From–To Analysis” function of the RESAS app (supported by Mobile Spatial Statistics of NTT DOCOMO^52^). The tile plot shows the OD flows at 2 pm in April 2016.

Figure 3 shows the inter-prefectural OD matrices at 2 pm on weekdays and weekends in April 2016. Each prefecture is numbered from 1 through to 47 (see Fig. 5 for the names of the prefectures). The color strengths in the tile plots represent the flow share. Panel (a) shows that most of the residents remained in their home prefectures. Panel (b) focuses on the OD matrix in the Greater Tokyo area (Saitama, Chiba, Tokyo, and Kanagawa). More than 10 percent of the residents in the neighboring prefectures of Tokyo were in Tokyo at 2 pm on weekdays during April 2016, but residents tended to remain in their home prefectures on weekends. Panels (b) of Fig. 3 shows the OD matrices in the Greater Osaka areas. Osaka also attracted people from neighboring prefectures, although the inflows were smaller than in the Greater Tokyo area.

**Figure 4:**
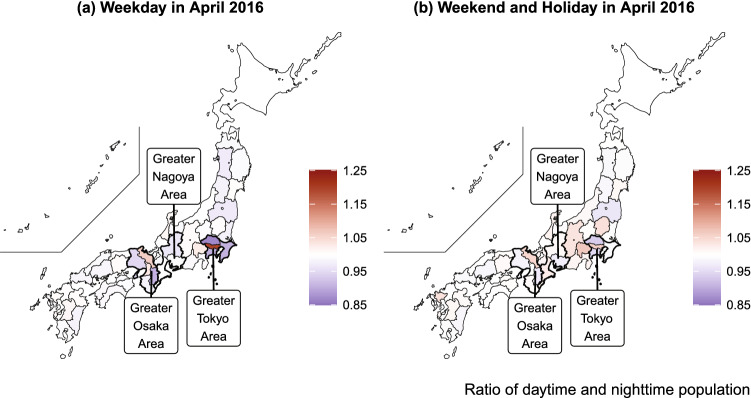
Ratio of daytime and nighttime populations on weekdays and at weekends in April 2016, created from the interregional mobility data obtained by the “From–To Analysis” function of the RESAS app (supported by Mobile Spatial Statistics of NTT DOCOMO^52^).

Figure 4 shows the ratio of daytime and nighttime populations by day of the week (weekdays versus weekends). As shown in Panel (a), people residing in the neighboring prefectures of Tokyo and Osaka tended to concentrate in Tokyo and Osaka, respectively, during the daytime on weekdays. However, Panel (b) shows that the spatial distribution of the population diverged across the country on weekends. The ratio of the daytime and nighttime population in rural resort areas near the Greater Tokyo area, such as Tochigi, Niigata, Yamanashi, Nagano, and Shizuoka, is greater than one, suggesting that these prefectures attract urban residents on holidays and weekends.

## Results

The simulations in this paper are based on the numbers of infectious individuals ${I}_{i}\left(t\right)$. All estimation results are available on the web application (see Supplementary Appendix S2 online).

**Figure 5:**
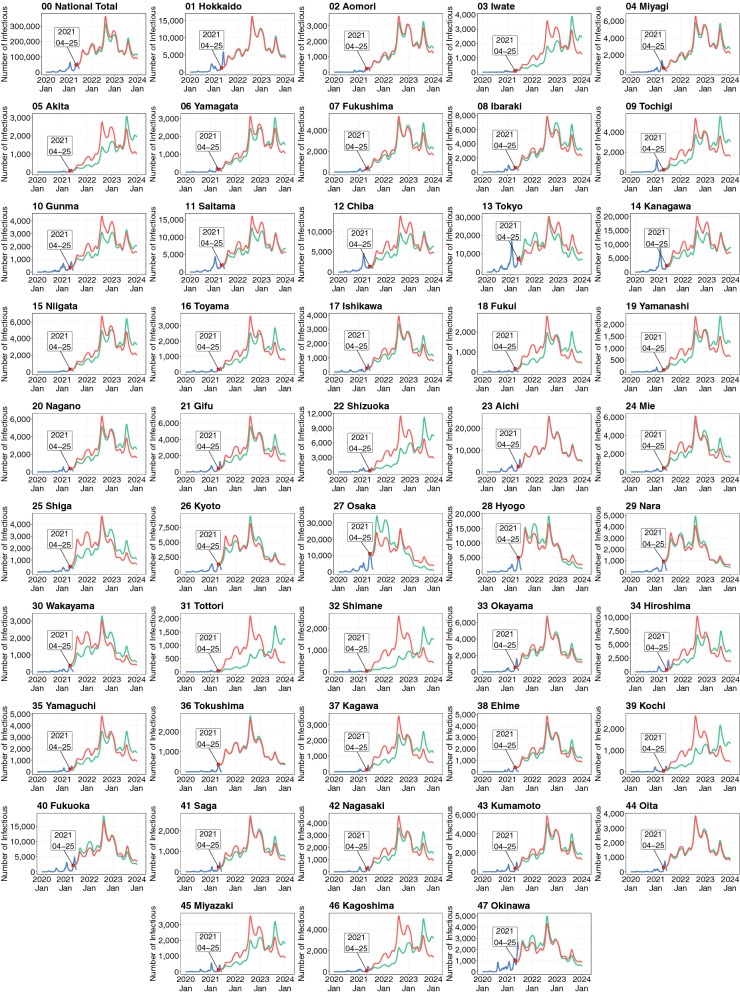
Simulated numbers of infectious persons by prefecture. Shown are the observed numbers of infectious people (blue lines) and the numbers of infectious people simulated by the spatial SEIR model with and without interregional mobility (red and green lines, respectively). The simulations were started on April 25, 2021.

Figure 5 shows the results of the baseline simulations. The simulated numbers of infectious individuals from the spatial SEIR model with and without interregional mobility (red and green lines, respectively) are shown, along with the observed number of infectious individuals up to April 25, 2021 (blue line).

The gap between the red and green lines captures the influence of interregional mobility on the spatial infection spread of COVID-19. This gap tended to be large in rural areas with lower numbers of infectious individuals at the start date of the simulation, such as Iwate, Akita, Yamagata, Fukushima, Ibaraki, Tochigi, Gunma, Niigata, Toyama, Fukui, Yamanashi, Nagano, Gifu, Shizuoka, Mie, Shiga, Wakayama, Tottori, Shimane, Hiroshima, Yamaguchi, Kagawa, Kochi, Nagasaki, Miyazaki, and Kagoshima. When interregional mobility was included, the numbers of infectious individuals simulated by the spatial SEIR model were more than twice those simulated without interregional mobility in Iwate, Akita, Shizuoka, Tottori, Shimane, Kochi, and Kagoshima, implying that interregional mobility caused the infection expansion in those prefectures.

In prefectures with large cities, such as Tokyo and Osaka, the spatial SEIR model with interregional mobility predicted lower numbers of infectious individuals than the model without interregional mobility. To understand why the interregional mobility generated these results, we calculated the ratios of the daytime and nighttime forces of infection in the Greater Tokyo, Nagoya, and Osaka Areas.

**Figure 6:**
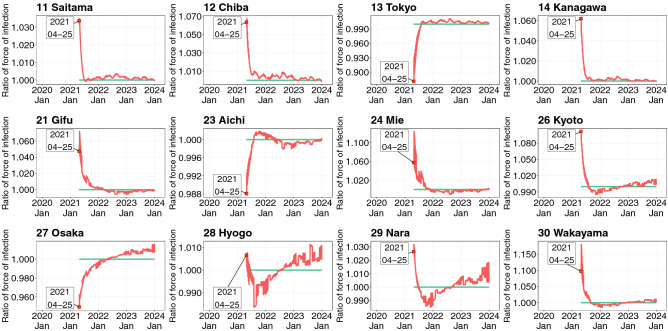
Ratio of daytime and nighttime force of infection by prefecture (see caption of Fig. 5 for details).

Figure 6 plots the ratios of the daytime and nighttime forces of infection simulated from the spatial SEIR model (see Supplementary Appendix S4 for other prefectures). Note that the ratio becomes one in the spatial SEIR model without interregional mobility, because the spatial population distributions during the daytime and nighttime are identical. As shown in Fig. 6, the ratios in Tokyo and Osaka were lower than one and gradually converged to one. Although large cities generally attract more external people than smaller cities and towns, the force of infection during the daytime decreased because the influx from neighboring prefectures contained susceptible individuals. The outflux of infectious individuals from Tokyo and Osaka also decreased the daytime force of infection. These results suggest that susceptible residents in Tokyo and Osaka were exposed to relatively low infection risks as a consequence of the interregional mobility.

Whether the outflux of infectious individuals from Tokyo and Osaka increased the daytime force of infection in neighboring prefectures (Saitama, Chiba, Kanagawa, Kyoto, Hyogo, Nara, and Wakayama) was also examined (see Supplementary Appendix S9 online). In the simulation, the interregional mobility of residents in Tokyo and Osaka was restricted, and residents from other prefectures were allowed to stay in Tokyo and Osaka in the daytime. The simulation results show that the force of infection decreased in neighboring prefectures (Supplementary Fig. S15). However, the quantitative influences of restricting the outflux of individuals from Tokyo and Osaka on preventing the spatial spread of infection were small (Supplementary Fig. S14). On the other hand, the daytime force of infection in Tokyo and Osaka remained lower than one, suggesting that the influx of susceptible individuals from the neighboring prefectures resulted in relatively low infection risks in Tokyo and Osaka.

Importantly, the total numbers of infectious individuals in Japan were almost identical in the SEIR models with and without interregional mobility. The simulation results showed that total numbers of infectious individuals simulated from the SEIR model without interregional mobility were slightly lower than those simulated from the SEIR model with interregional mobility until April 2023, but this relationship reversed around May 2023, suggesting that restricting the interregional mobility slowed the speed of the infection spread but did not effectively reduce the overall infection expansion.

**Figure 7:**
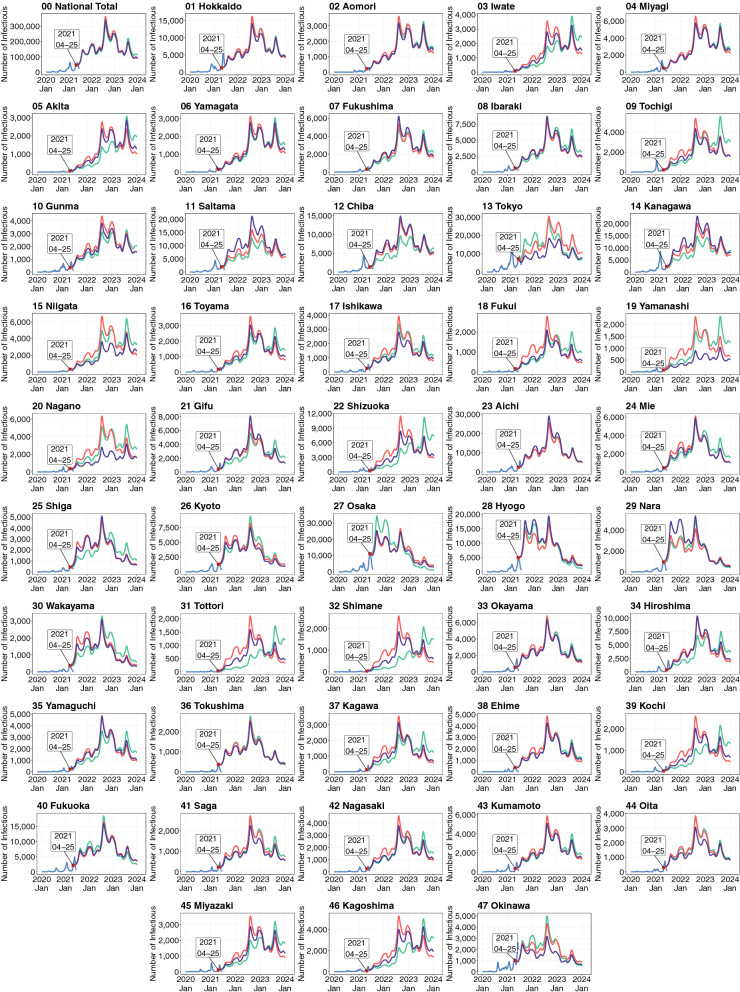
Simulated numbers of infectious people by prefecture under the assumptions that the government either does or does not restrict the interregional mobility of infectious people to remain in their residential prefectures. Shown are the observed numbers of infectious people up to April 25, 2021 (blue lines), the numbers of infectious people simulated by the spatial SEIR model without interregional mobility (green lines), and those simulated by the spatial SEIR model with interregional mobility, assuming that the interregional mobility of only infectious people is restricted (purple lines) or that free mobility across prefectures is allowed for all individuals (red lines). The simulations were started on April 25, 2021.

Figure 7 shows the simulation results of the extended spatial SEIR model in a situation where infectious persons were restricted to their residential prefectures, whereas susceptible, exposed (infected but not yet infectious), and recovered people could commute freely or travel across prefectures (see Supplementary Appendix S6 for the ratio of daytime and nighttime force of infection). In other words, the OD matrix for infectious persons becomes a diagonal matrix, whereas the OD matrix for susceptible, exposed, and recovered people is taken from the mobility data.

The simulation results showed that the interregional mobility restriction for infectious persons did not reduce the total number of COVID-19 infections in Japan but affected the pattern of spatial spread. For example, this restriction reduced the number of new infections in rural resort areas, such as Tochigi, Niigata, Yamanashi, Nagano, and Shizuoka, where the ratio of the daytime and nighttime population in these regions on holidays and weekends was greater than one.

Interregional mobility of susceptible people also plays a key role in explaining the spatial spread of COVID-19 infections. In the Greater Tokyo Areas, susceptible people in Saitama, Chiba, and Kanagawa commute daily to Tokyo in the daytime. They are exposed to infection risk during the day, such as face-to-face meetings and eating in the cafeteria. Infections in different locations in the daytime cause spatial spread to their residential communities. The simulation results in Fig. 7 also show that restricting commuting and traveling to prefectures with many infectious people helps delay the geographical expansion of infections from urban to rural prefectures.

The Supplementary Information provides additional simulation results. As a robustness check, we conducted a validity check of the long-term impact of interregional mobility on the spatial spread of infection, comparing the observed numbers of infections (Supplementary Appendix S3). The simulation results in Supplementary Fig. S2 starting from the first declaration of the state of emergency on April 7, 2020 showed that the interregional mobility accelerated the geographical expansion of infection from urban prefectures with many infectious people (e.g., Greater Tokyo and Osaka areas) to rural prefectures (e.g., Aomori, Iwate, Akita, Tochigi, Niigata, Shizuoka, Mie, Tottori, Shimane, Okayama, Tokushima, Kagawa, Nagasaki, and Kagoshima).

Furthermore, we used the OD matrix based on the locations where people resided and where they were located at 8 pm to consider the higher infection risk in bars and restaurants in the nighttime (Supplementary Appendix S5). The simulation results in Supplementary Fig. S6 only showed slight changes in the spatial spread of COVID-19 infection from those obtained from the interregional mobility based on locations where people are located at 2 pm, suggesting that many people continue to stay in the locations where they were at 2 pm at the prefecture level.

To discuss effective policies measures of the interregional mobility restriction, we evaluated how the restriction of interregional mobility only for the Greater Tokyo and Osaka areas affected the spatial spread of infection (Supplementary Appendices S7 and S8). It was assumed that residents in the Greater Tokyo and Osaka areas are restricted to remain in each prefecture, and residents in other prefectures are allowed to commute and travel across prefectures, except the Greater Tokyo and Osaka area. The simulation results in Supplementary Figs. S10 and S12 showed that interregional mobility restrictions only for Greater Tokyo and Osaka areas helped prevent the infection from spreading to neighboring prefectures. Distant prefectures that were connected to the Greater Tokyo and Osaka areas also reduced the infection expansion through restrictions of interregional mobility only for the Greater Tokyo and Osaka areas, suggesting that monitoring daily data on interregional mobility with a direction can help policymakers detect future outbreaks of COVID-19.

## Discussion

The spatial SEIR model with interregional mobility revealed the effect of interregional mobility on the geographical infection spread of COVID-19. The simulations relied on past interregional mobility data obtained from the locational information of mobile phone users. By analyzing these data, we could evaluate the differences in interregional mobility by season, day of the week, and period of the day. The government of Japan has recognized the effectiveness of high-frequency and real-time mobility data in mitigating the COVID-19 pandemic^13^. In this study, the interregional mobility data were incorporated into a daily OD matrix, showing the applicability of recent geospatial information technology in epidemic models.

From the simulation results, we can understand two important aspects of control measures. The first aspect involves the spatial spread of COVID-19. As expected, restricting the interregional mobility prevent the wide geographical spreading of the infection. This control measure is especially important for rural regions with scarce healthcare resources. Consistent with our simulation results, college student travel during the spring break was found to contribute to local infection transmission in the U.S^53^.

Surprisingly, in prefectures with large cities that attract outside workers (such as Tokyo and Osaka), the number of infections increased after restricting interregional mobility. Although restricting mobility has reduced the total number of COVID-19 cases per capita in some U.S. cities^54^, our simulation results from the spatial SEIR model suggest that the interregional mobility restriction has heterogeneous impacts on the infection expansion across regions in Japan. For example, an influx of uninfected persons from outside reduces the infection risk in the daytime in those regions. Therefore, restricting interregional mobility without restricting intraregional mobility will result in an increase in the infection risk to residents in large cities.

The second aspect of control measures is whether interregional mobility restrictions can reduce the national total number of infections. Distinguishing between the short- and long-term control measures is important, and the fundamental goal should be toward reducing the overall epidemic size^22^. According to our simulation results, solely restricting interregional mobility has a limited effect on reducing the national total number of infectious individuals. Imposing interregional mobility restrictions cuts the growth rate in national total number of infectious individuals only when the effective reproduction number is one or higher. Moreover, research in the UK indicates that regional lockdowns effectively reduced the overall epidemic size only when the transmission rate remains persistently low^22^. These results imply that the efficacy of imposing interregional mobility restrictions (in terms of reducing the overall epidemic size) is very sensitive to the timing of the restriction.

This study has some theoretical and empirical limitations. First, this study did not consider the effect of vaccination and the appearance of new variants in this long-run simulation. It is expected that COVID-19 herd immunity is achieved through vaccinations. However, it is still unclear whether COVID-19 vaccines protect people from getting infected or from spreading the virus to others^55^. In addition, new variants of SARS-CoV-2 that are more transmissible and resistant to current vaccines may emerge while vaccination is proceeding. Therefore, this study did not consider factors related to vaccination and new variants in our simulations.

Second, the study did not consider difference in infection risks during the process of commuting and traveling despite some simulated and empirical studies on the infection risk in public transportation^23,56–59^. In Japan, public transportation in metropolitan areas by trains, metro, and buses supports mass mobility, implying that commuters in urban areas are exposed to higher infection risks than commuters by car in rural areas. Long-distance travelers tend to travel by airplane, where the infection risk will increase if social distancing is difficult. However, clearly determining which type of public transportation shows the highest infection risk is difficult because the risk depends on congestion and seat location, which vary by time of day, and thus this study did not explicitly consider infection risk in the theoretical model. In the future, such empirical results should be incorporated into the theoretical framework.

Third, this study relied on 2016 mobility data based on the Mobile Spatial Statistics (NTT DOCOMO) because of data availability instead of the latest mobility data in 2020–2021. Although it was assumed that inter-prefectural mobility patterns were unchanged as a counterfactual setting, it is also important to investigate how the COVID-19 pandemic caused behavioral changes in interregional mobility. For example, strong NPIs in urban areas, such as the closure of bars, restaurants, and shopping malls, may incentivize travel to rural areas, where the infection risk is low. In turn, behavioral changes in interregional mobility simultaneously affect the expansion of COVID-19 infections. Further, commute or travel decisions should be described endogenously in the theoretical model.

Owing to some theoretical and empirical limitations, this study focused on the qualitative aspects of the spatial spread of COVID-19 infections through interregional mobility restrictions and found that the force of infection fluctuated between daytime and nighttime because of interregional mobility; this was also demonstrated by the interregional mobility data. Therefore, this study provides an interesting application using interregional mobility data obtained by recent geospatial information technology, and important pointers as to how mobility data might be incorporated into future epidemiological research on infectious diseases.

In conclusion, interregional mobility restrictions dominantly affect the spatial distribution of infection and the speed of the infection spread but play a limited role in reducing the national total number of infections because urban areas with many infectious people are exposed to a higher force of infection when the interregional mobility is restricted. To reduce the epidemic size, the intraregional mobility also should be restricted within urban areas in combination with interregional mobility restrictions. Promoting additional NPIs, such as case isolation in the home, self-quarantine, remote work, and restriction of mass gatherings, should be implemented^4,5,23^. The most important implication of restricting interregional mobility is the avoidance of an epidemic peak that overwhelms the existing healthcare services. In Japan, healthcare resources are typically concentrated in urban areas and scarce in rural areas^60^. In that sense, restricting interregional mobility is an effective control measure in preventing the geographical expansion of infection.

## Supplementary Information

Supplementary Information.

## Data Availability

The R code and data for this study are available on GitHub (https://github.com/keisukekondokk/spatial-seir).
