# Supplementary material for: Simulating the impacts of interregional mobility restriction on the spatial spread of COVID-19 in Japan
Source: Sci Rep. 2021 Sep 23;11:18951. doi: 10.1038/s41598-021-97170-1 (PMC8460743; doi:10.1038/s41598-021-97170-1)
Supplement: Supplementary file 1 — Supplementary Information. [file 41598_2021_97170_MOESM1_ESM.pdf]

Supplementary Information

Simulating the Impacts of Interregional Mobility Restriction on the Spatial Spread of COVID-19 in Japan

Keisuke Kondo*

RIETI

This Supplementary Information provides additional simulation results. The second and forth scenarios were discussed in the main text.

*Research Institute of Economy, Trade and Industry (RIETI). 1-3-1 Kasumigaseki, Chiyoda-ku, Tokyo, 100–8901, Japan. (e-mail: kondo-keisuke@rieti.go.jp).

Contents

Appendix S1 Declaration of state of emergency 3
Appendix S2 COVID-19 simulator 6
Appendix S3 Case 1: Validity check of the long-run impacts of interregional mobility restriction 8
Appendix S4 Case 2: Projecting the long-run impacts of interregional mobility restriction 13
Appendix S5 Case 3: Comparison of interregional mobility at 2 pm and 8 pm 18
Appendix S6 Case 4: Interregional mobility restriction only for infectious people 23
Appendix S7 Case 5: Interregional mobility restriction only for the Greater Tokyo area 28
Appendix S8 Case 6: Interregional mobility restriction only for the Greater Osaka area 33
Appendix S9 Case 7: Interregional mobility restriction only for Tokyo and Osaka 38

Appendix S1 Declaration of state of emergency

Table S1 presents the duration of the declarations of a state of emergency in each prefecture in Japan according to the information provided by the national government of Japan (as of July 27, 2021).[1] At first, a state of emergency was declared on April 7, 2020, for seven prefectures (Saitama, Chiba, Tokyo, Kanagawa, Osaka, Hyogo, and Fukuoka), and it was extended to all 47 prefectures on April 16, 2020. The first state of emergency ended in 39 prefectures on May 14, 2020; in Kyoto, Osaka, and Hyogo on May 21, 2020; and finally in Hokkaido, Saitama, Chiba, Tokyo, and Kanagawa on May 25, 2020.

The second declaration of a state of emergency included the Greater Tokyo area (Saitama, Chiba, Tokyo, and Kanagawa) on January 7, 2021 (which came into effect on January 8, 2021), and it was extended to the Greater Osaka area (Kyoto, Osaka, and Hyogo), Tochigi, Aichi, Gifu, and Fukuoka on January 13, 2021 (which came into effect on January 14, 2021). The second state of emergency ended in Tochigi on February 7, 2021; in Gifu, Aichi, Kyoto, Osaka, Hyogo, and Fukuoka on February 28, 2021; and finally in Saitama, Chiba, Tokyo, and Kanagawa on March 21, 2021.

The third declaration came into effect on April 25, 2021, for Tokyo and the Greater Osaka area (Kyoto, Osaka, and Hyogo), and it was extended to Aichi and Fukuoka on May 12, 2021; to Hokkaido, Okayama, and Hiroshima on May 16, 2021; and to Okinawa on May 23, 2021. This state of emergency ended in nine of the aforementioned prefectures, with the exception of Okinawa, on June 20, 2021. However, a new declaration became effective, again, in Tokyo from July 12, 2021. As of July 27, 2021, Tokyo and Okinawa remain in a state of emergency.

Prefecture Code	Prefecture Name	Start Date	End Date
1	Hokkaido	April 16, 2020	May 25, 2020
		May 16, 2021	June 20, 2021
2	Aomori	April 16, 2020	May 14, 2020
3	Iwate	April 16, 2020	May 14, 2020
4	Miyagi	April 16, 2020	May 14, 2020
5	Akita	April 16, 2020	May 14, 2020
6	Yamagata	April 16, 2020	May 14, 2020
7	Fukushima	April 16, 2020	May 14, 2020
8	Ibaraki	April 16, 2020	May 14, 2020
9	Tochigi	April 16, 2020	May 14, 2020
		January 14, 2021	February 07, 2021
10	Gunma	April 16, 2020	May 14, 2020
11	Saitama	April 07, 2020	May 25, 2020
		January 08, 2021	March 21, 2021
12	Chiba	April 07, 2020	May 25, 2020
		January 08, 2021	March 21, 2021
13	Tokyo	April 07, 2020	May 25, 2020
		January 08, 2021	March 21, 2021
		April 25, 2021	June 20, 2021
		July 12, 2021	August 22, 2021 (scheduled)
14	Kanagawa	April 07, 2020	May 25, 2020
		January 08, 2021	March 21, 2021
15	Niigata	April 16, 2020	May 14, 2020
16	Toyama	April 16, 2020	May 14, 2020
17	Ishikawa	April 16, 2020	May 14, 2020
18	Fukui	April 16, 2020	May 14, 2020
19	Yamanashi	April 16, 2020	May 14, 2020
20	Nagano	April 16, 2020	May 14, 2020
21	Gifu	April 16, 2020	May 14, 2020
		January 14, 2021	February 28, 2021
22	Shizuoka	April 16, 2020	May 14, 2020
23	Aichi	April 16, 2020	May 14, 2020
		January 14, 2021	February 28, 2021
		May 12, 2021	June 20, 2021
24	Mie	April 16, 2020	May 14, 2020

Table S1. Effective dates of declarations of a state of emergency by prefecture as of July 27, 2021.

Prefecture Code	Prefecture Name	Start Date	End Date
25	Shiga	April 16, 2020	May 14, 2020
26	Kyoto	April 16, 2020	May 21, 2020
		January 14, 2021	February 28, 2021
		April 25, 2021	June 20, 2021
27	Osaka	April 07, 2020	May 21, 2020
		January 14, 2021	February 28, 2021
		April 25, 2021	June 20, 2021
28	Hyogo	April 07, 2020	May 21, 2020
		January 14, 2021	February 28, 2021
		April 25, 2021	June 20, 2021
29	Nara	April 16, 2020	May 14, 2020
30	Wakayama	April 16, 2020	May 14, 2020
31	Tottori	April 16, 2020	May 14, 2020
32	Shimane	April 16, 2020	May 14, 2020
33	Okayama	April 16, 2020	May 14, 2020
		May 16, 2021	June 20, 2021
34	Hiroshima	April 16, 2020	May 14, 2020
		May 16, 2021	June 20, 2021
35	Yamaguchi	April 16, 2020	May 14, 2020
36	Tokushima	April 16, 2020	May 14, 2020
37	Kagawa	April 16, 2020	May 14, 2020
38	Ehime	April 16, 2020	May 14, 2020
39	Kochi	April 16, 2020	May 14, 2020
40	Fukuoka	April 07, 2020	May 14, 2020
		January 14, 2021	February 28, 2021
		May 12, 2021	June 20, 2021
41	Saga	April 16, 2020	May 14, 2020
42	Nagasaki	April 16, 2020	May 14, 2020
43	Kumamoto	April 16, 2020	May 14, 2020
44	Oita	April 16, 2020	May 14, 2020
45	Miyazaki	April 16, 2020	May 14, 2020
46	Kagoshima	April 16, 2020	May 14, 2020
47	Okinawa	April 16, 2020	May 14, 2020
		May 23, 2021	August 22, 2021 (scheduled)

Table S1. Effective dates of declarations of a state of emergency by prefecture as of July 27, 2021 (*continued*).

Appendix S2 COVID-19 simulator

This study developed a Shiny application to visualize the simulated number of the susceptible, exposed, infectious, and recovered individuals in each case scenario. All simulation results are provided on the web application.

(URL: `https://keisuke-kondo.shinyapps.io/covid19-simulator-japan/`).

Figure S1 shows the inter-prefectural flows on the web application (Click Spatial Network Data on the Visualization menu). The width of the line and the strength of the line color represent the size of the flow. The map shows the share of people residing in each prefecture who stayed in Tokyo at 2 pm on a weekday in April 2016. For example, 15–20 % of people residing in Saitama stayed in Tokyo at 2 pm on a weekday in April 2016. All bilateral flows across the 47 prefectures were visualized on the web application.

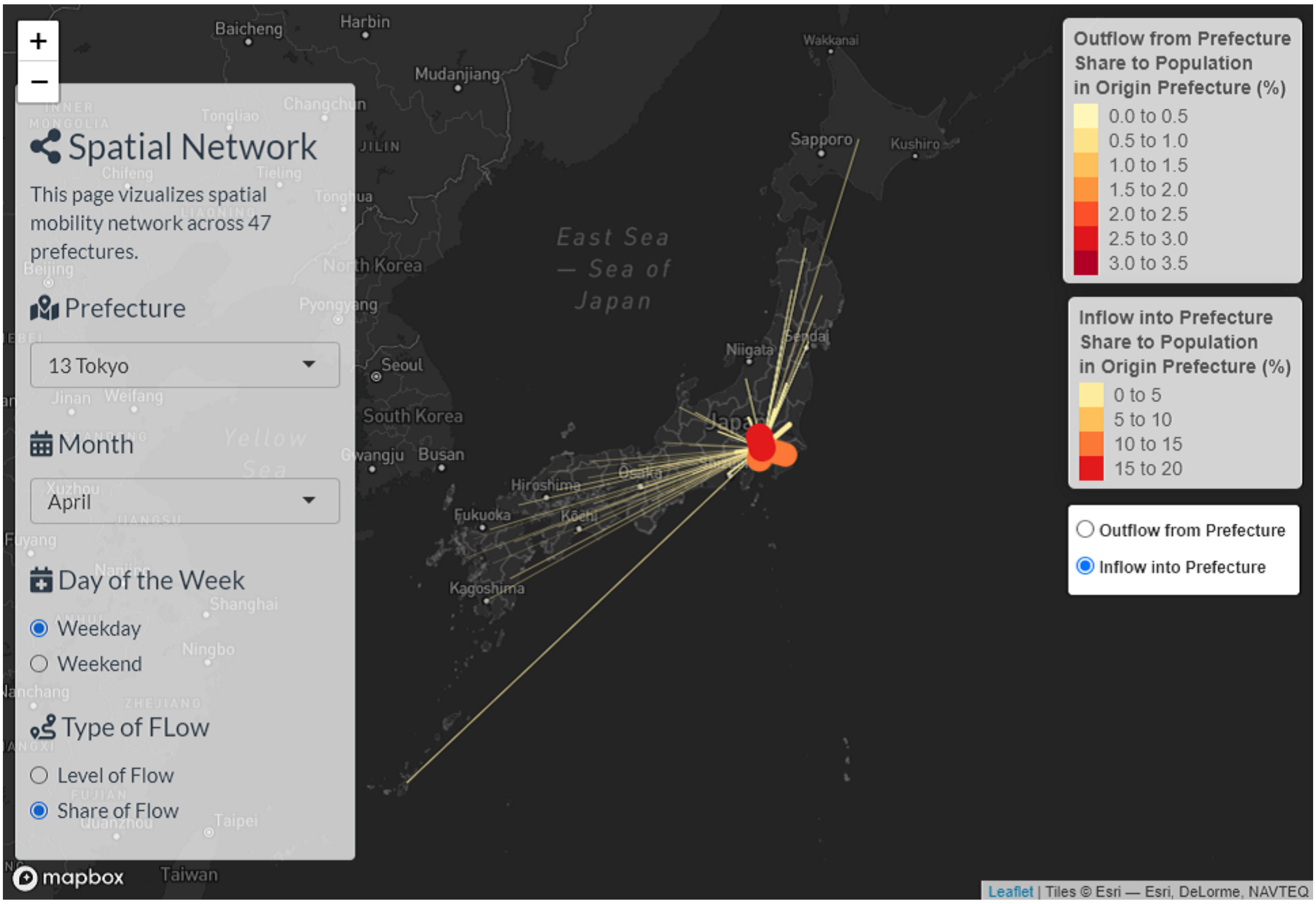

Figure S1. Interregional mobility across the 47 prefectures, constructed from the interregional mobility data obtained by the "From–To Analysis" function of the RESAS app (supported by Mobile Spatial Statistics of NTT DOCOMO). The map shows the share of people residing in each prefecture who stayed in Tokyo at 2 pm on weekday in April 2016. For example, 15–20 % of people residing in Saitama stayed in Tokyo at 2 pm on weekday in April 2016.

Appendix S3 Case 1: Validity check of the long-run impacts of interregional mobility restriction

Figures S2 and S3 show the simulation results starting from the first declaration of the state of emergency on April 7, 2020. Case 1 compares two counterfactual scenarios. The first is that interregional mobility is allowed without any restrictions in the long run. The second is that mobility across prefectures is restricted in the long run.

The simulation results showed that the interregional mobility accelerated the geographical expansion of infection from urban prefectures with many infectious people to rural prefectures, such as Aomori, Iwate, Akita, Tochigi, Niigata, Shizuoka, Mie, Tottori, Shimane, Okayama, Tokushima, Kagawa, Nagasaki, and Kagoshima. The restriction of interregional mobility prevented the influx of SARS-CoV-2 into these rural prefectures. However, the restriction of interregional mobility was not sufficient to reduce the total number of new infections in Japan in the long run.

Interregional mobility restrictions dominantly affect the spatial distribution of infection and the speed of the infection spread but play a limited role in reducing the national total number of infections. To reduce the epidemic size, the intraregional mobility also should be restricted within urban areas in combination with interregional mobility restrictions. Promoting other NPIs, such as case isolation in the home, self-quarantine, remote work, and restriction of mass gatherings, should be implemented.[2–4]

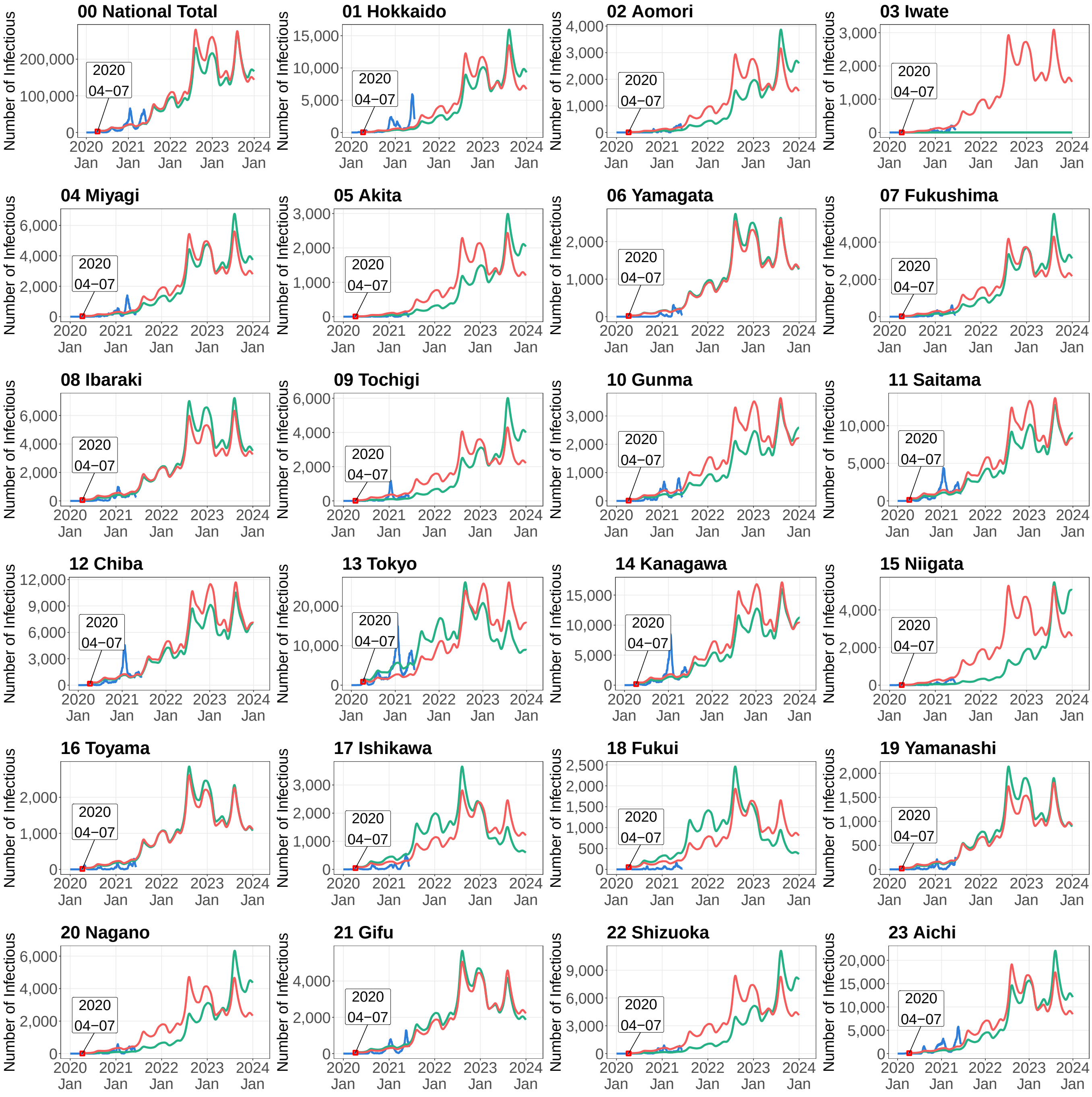

Figure S2. Simulated numbers of infectious people by prefecture in case 1. Shown are the observed numbers of infectious people (blue lines) and the numbers of infectious people simulated by the spatial SEIR model with and without interregional mobility (red and green lines, respectively).

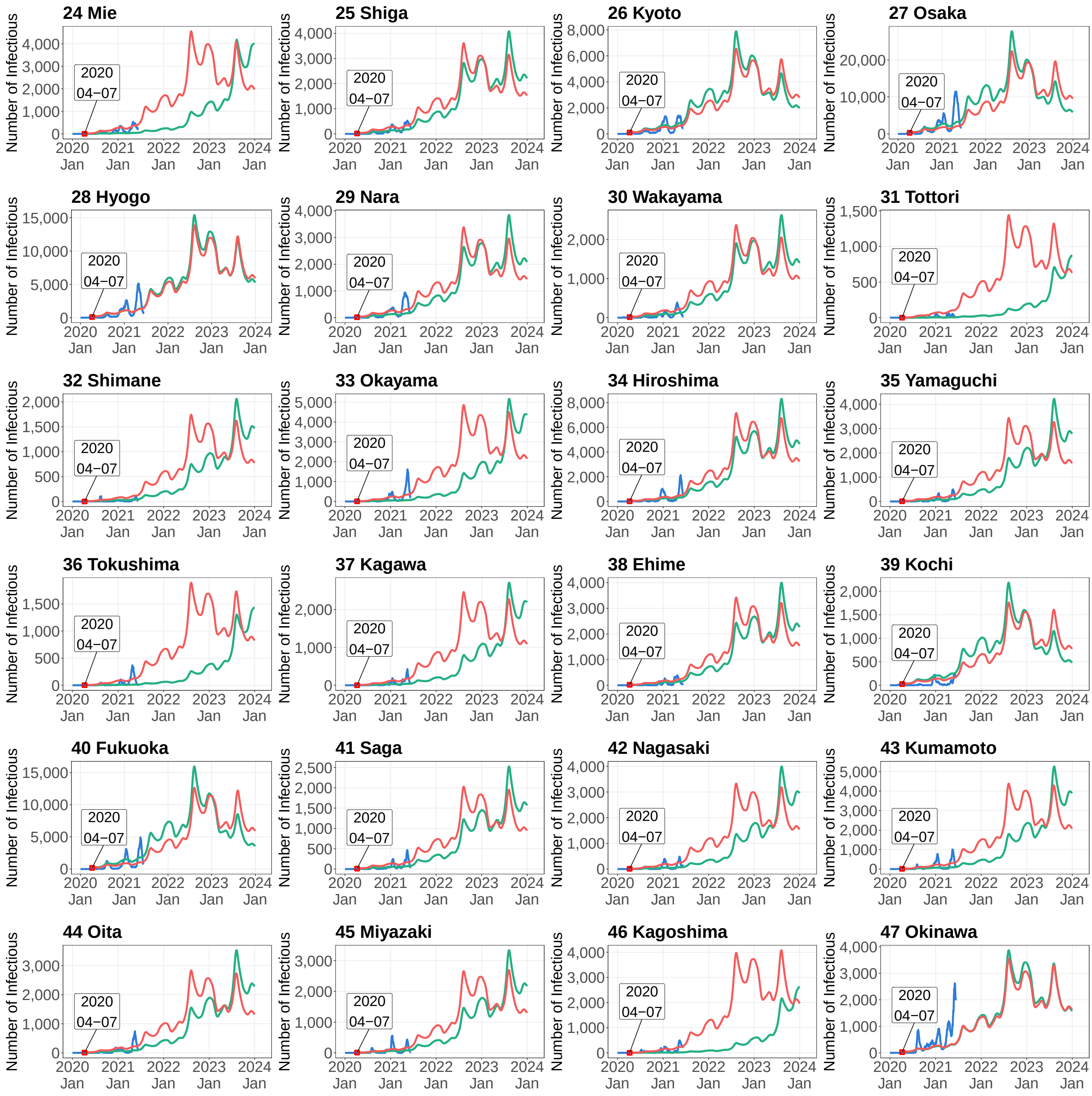

Figure S2. Simulated numbers of infectious people by prefecture in case 1 (*continued*).

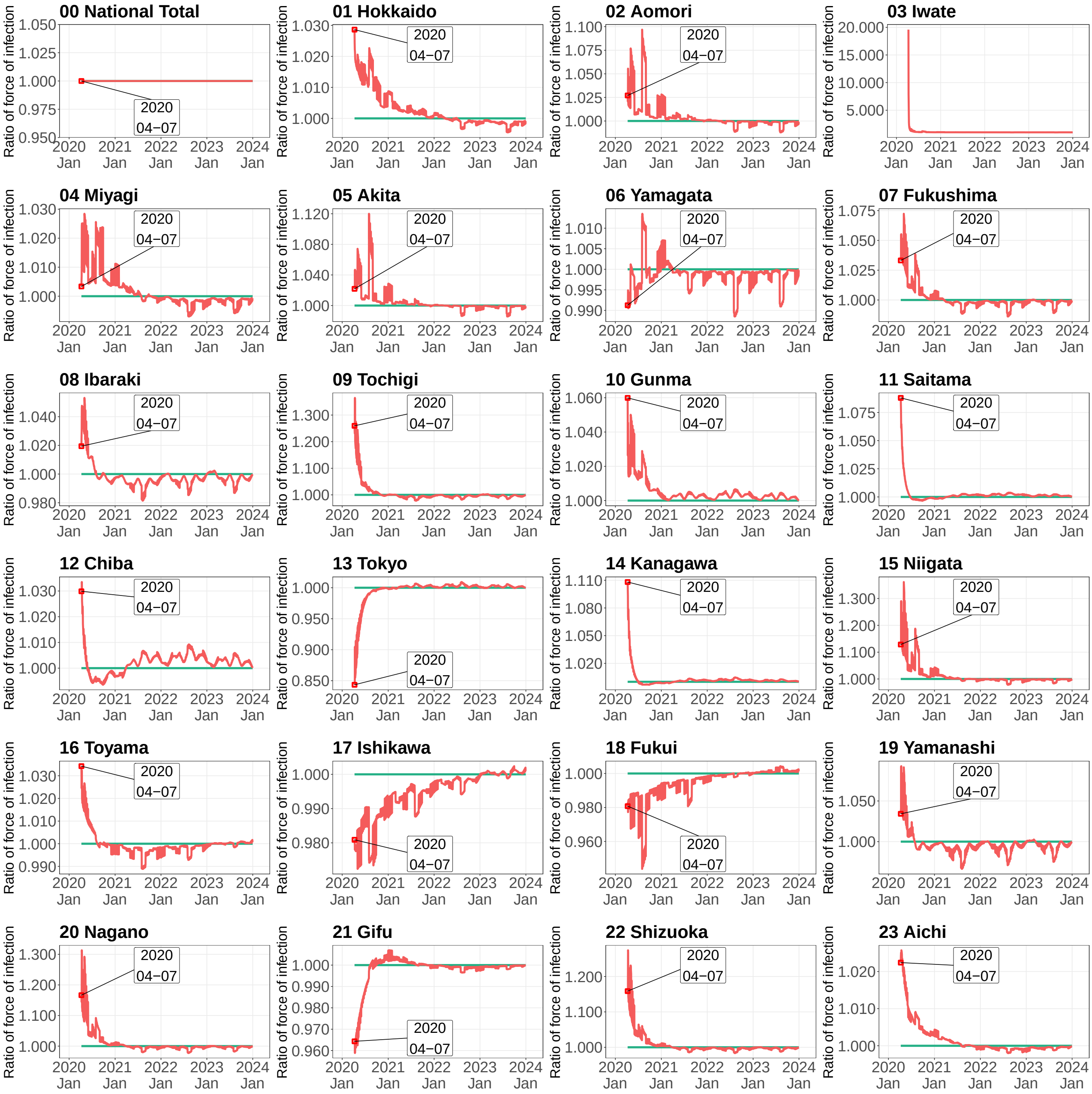

Figure S3. Ratio of daytime and nighttime force of infection by prefecture in case 1. Shown are the ratios of daytime and nighttime force of infection in the spatial SEIR model with and without interregional mobility (red and green lines, respectively).

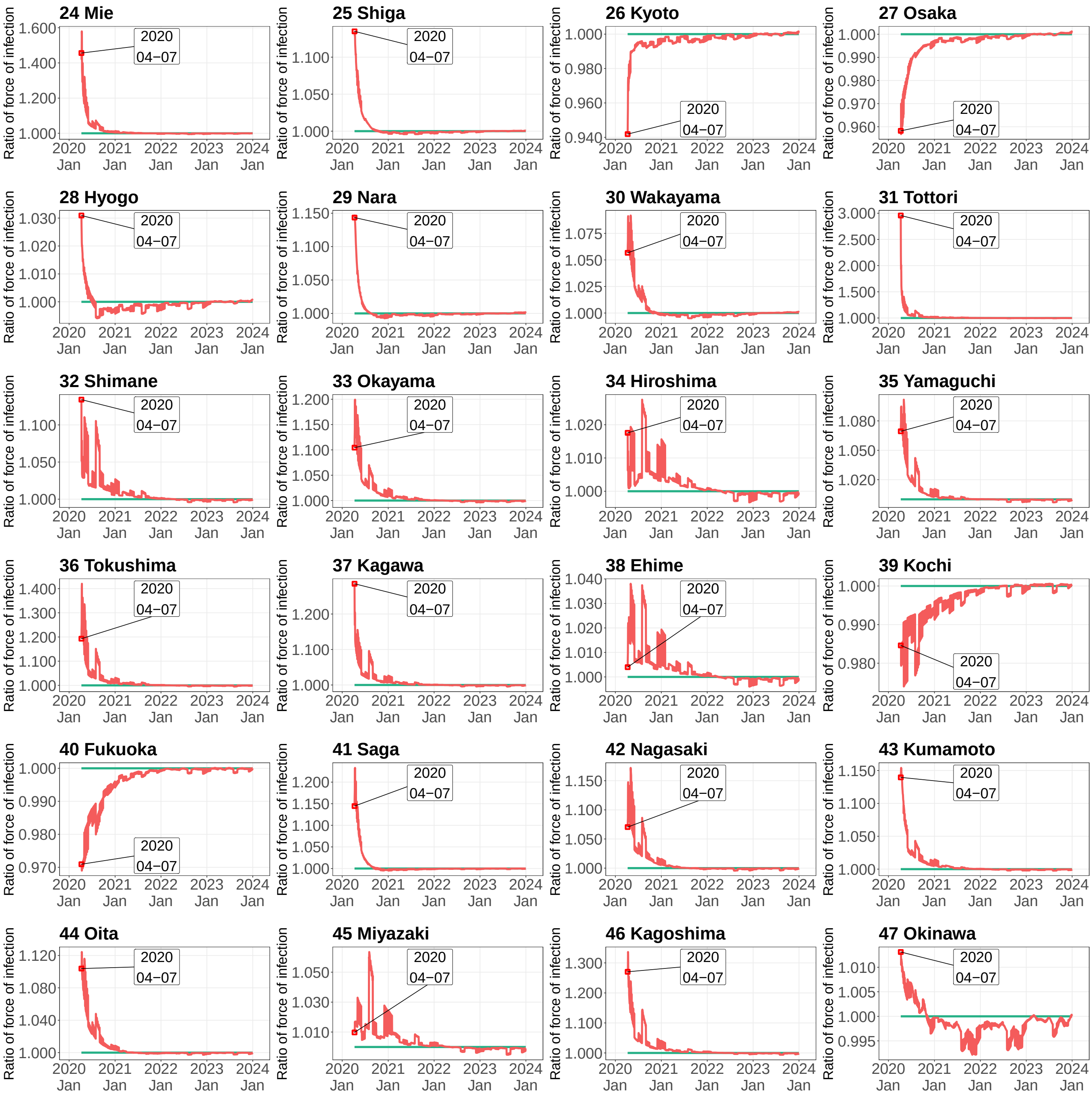

Figure S3. Ratio of daytime and nighttime force of infection by prefecture in case 1 (*continued*).

Appendix S4 Case 2: Projecting the long-run impacts of interregional mobility restriction

Figures S4 and S5 present the simulation results in the case where interregional mobility is restricted from April 25, 2021 (two days before the third declaration of a state of emergency in Tokyo, Kyoto, Osaka, and Hyogo). Case 2 simulates the long-run impacts of interregional mobility on the spatial spread of COVID-19 infection based on the spatial SEIR models with and without interregional mobility. Case 2 was discussed in the main text.

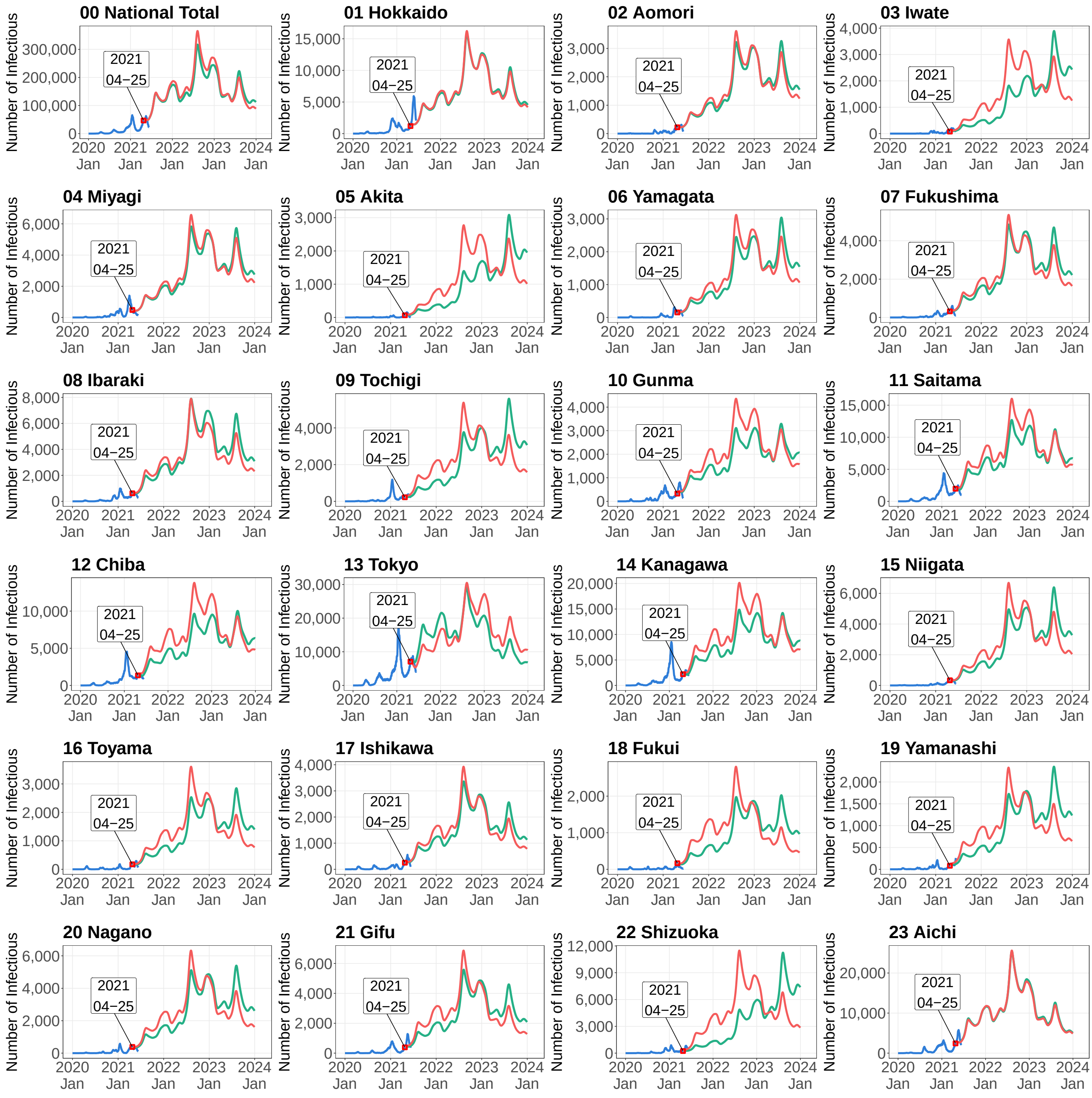

Figure S4. Simulated numbers of infectious people by prefecture in case 2. Shown are the observed numbers of infectious people (blue lines), the numbers of infectious people simulated by the spatial SEIR model without interregional mobility (green lines), and those simulated by the spatial SEIR model with interregional mobility (red lines).

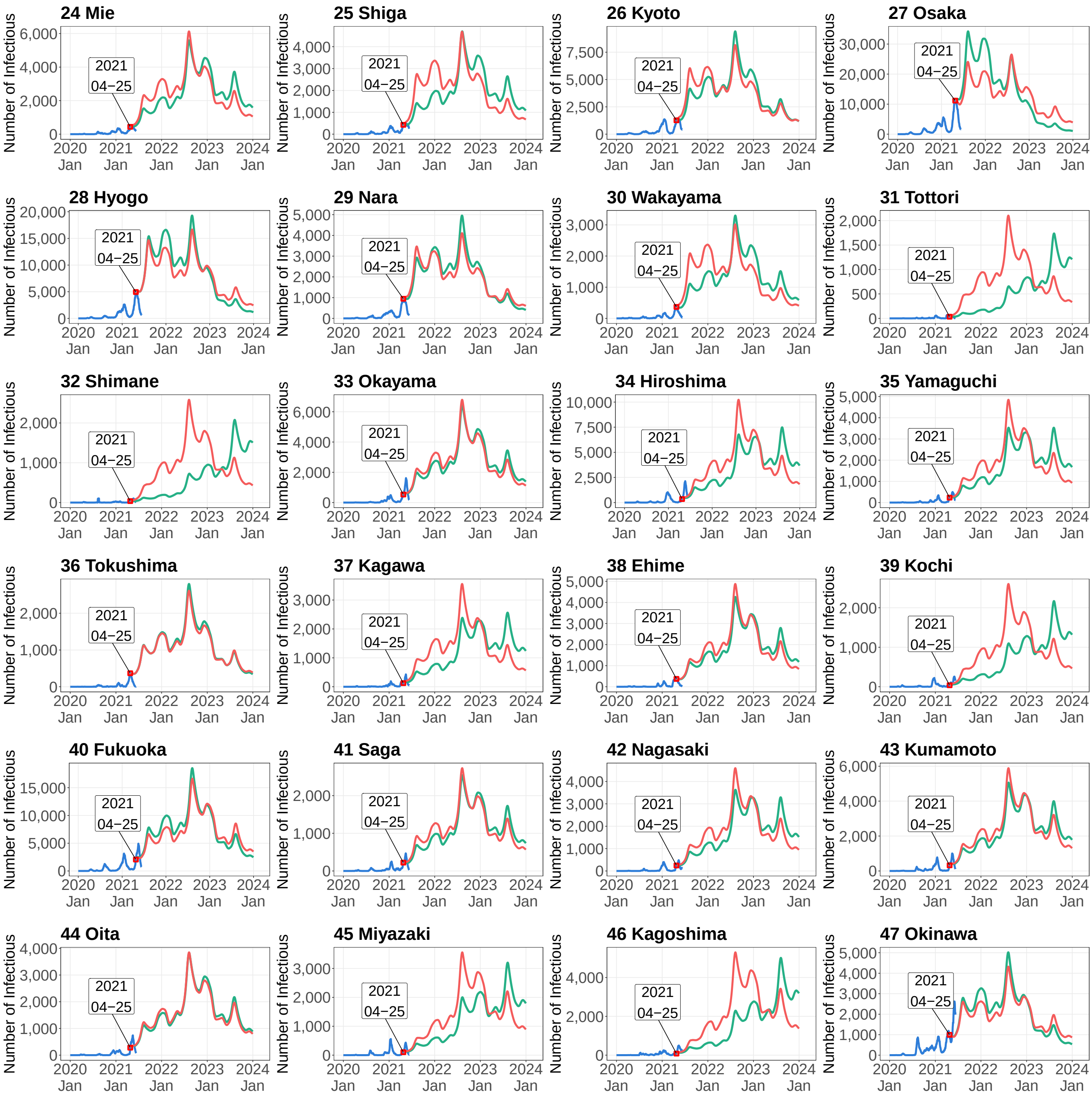

Figure S4. Simulated numbers of infectious people by prefecture in case 2 (*continued*).

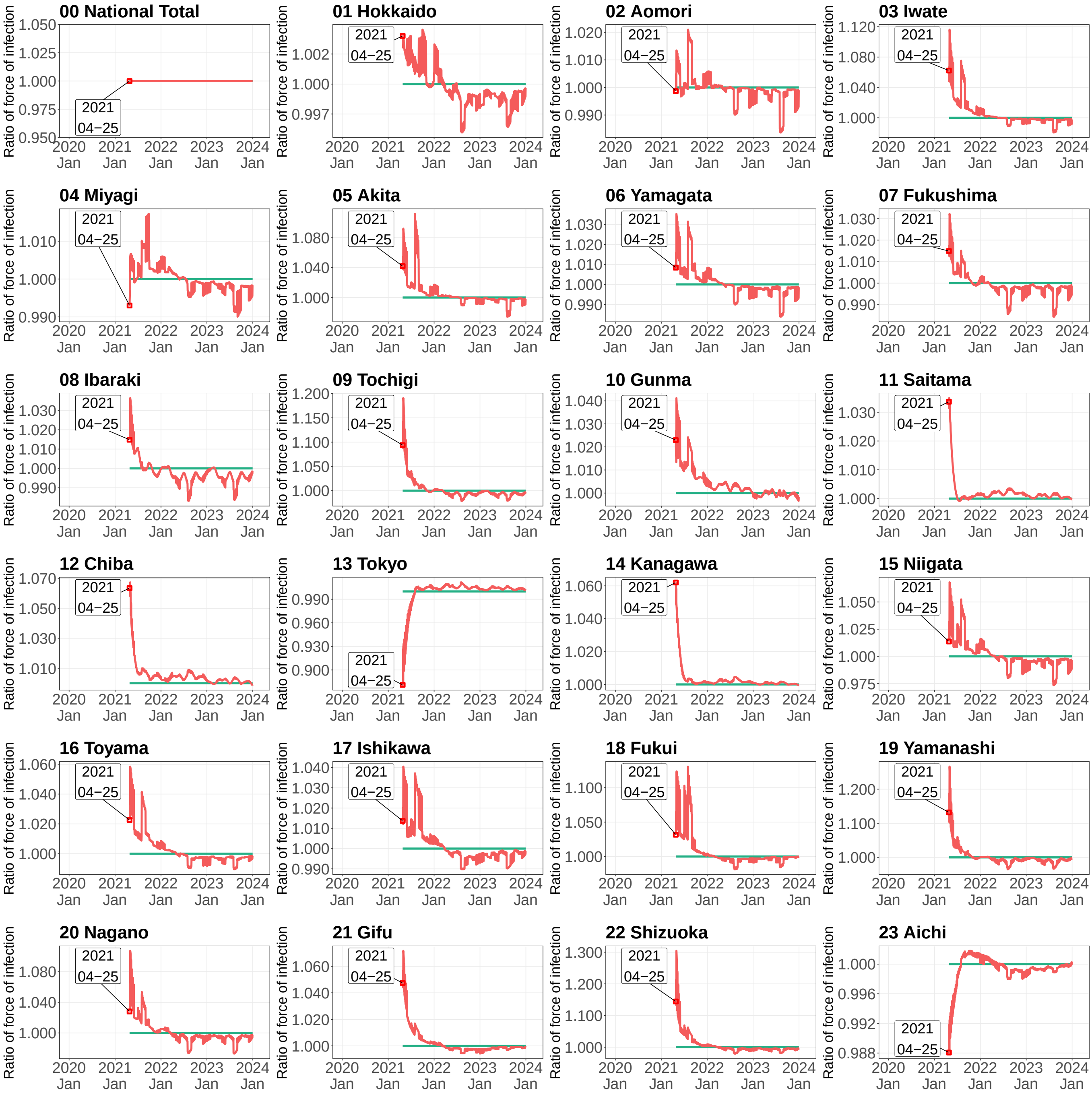

Figure S5. Ratio of daytime and nighttime force of infection by prefecture in case 2. Shown are the ratios of daytime and nighttime force of infection in the spatial SEIR model with and without interregional mobility (red and green lines, respectively).

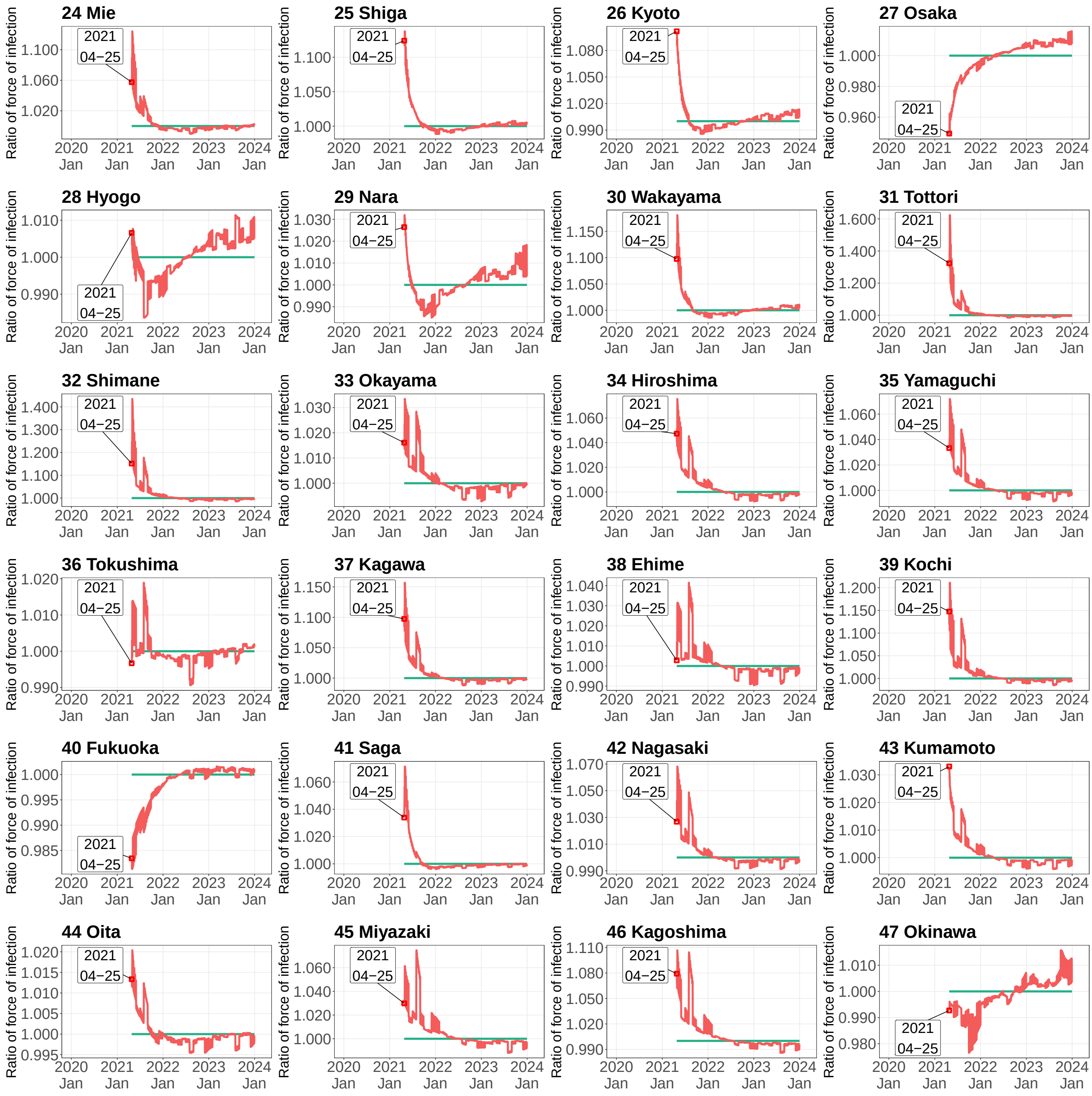

Figure S5. Ratio of daytime and nighttime force of infection by prefecture in case 2 (*continued*).

Appendix S5 Case 3: Comparison of interregional mobility at 2 pm and 8 pm

Figures S6 and S7 show the simulation results of the spatial SEIR model based on different patterns of interregional mobility to consider the higher infection risk in bars and restaurants at night time. Case 3 considers the origin–destination (OD) matrix based on where they reside and where people are located at 8 pm. The simulation results only showed slight changes in the spatial spread of COVID-19 infection from those obtained from the interregional mobility based on locations where people are located at 2 pm, suggesting that many people continue to stay in the locations where they were at 2 pm at the prefecture level.

Although this study theoretically assumes that people are exposed to infection in the location where they stay in the daytime, the mobility data based on the locations at 2 pm partly capture the locational factors of the infection in the nighttime. However, considering commuting or the travel route and location history during the day is essential if the spatial spread of the infection is considered at a small geographical unit, such as municipality and county.

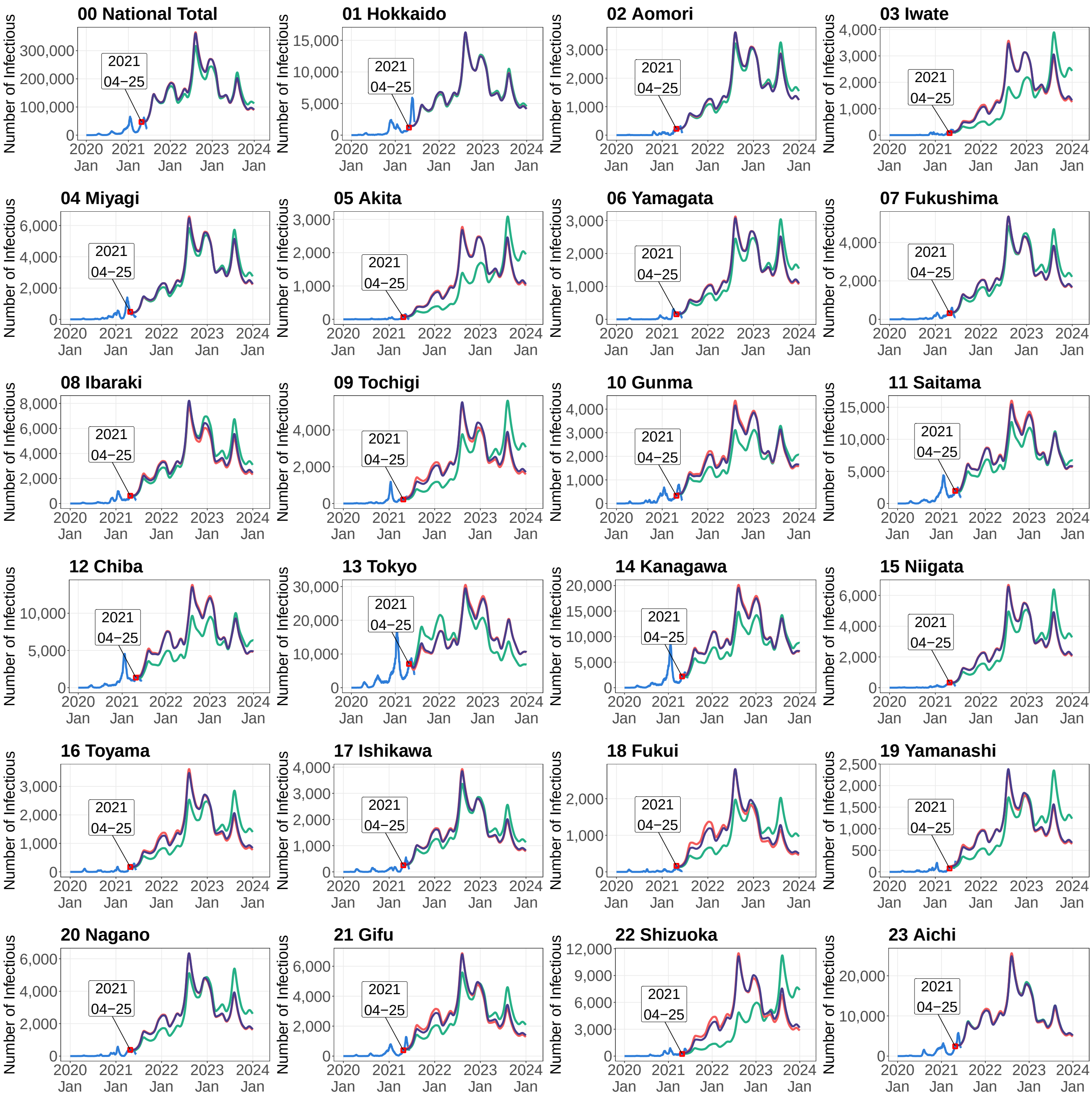

Figure S6. Simulated numbers of infectious people by prefecture in case 3. Shown are the observed numbers of infectious people (blue lines), the numbers of infectious people simulated by the spatial SEIR model without interregional mobility (green lines), and those simulated by the spatial SEIR model with interregional mobility. The interregional mobility pattern is based on locations where people are located at 2 pm and 8 pm (red and purple lines, respectively).

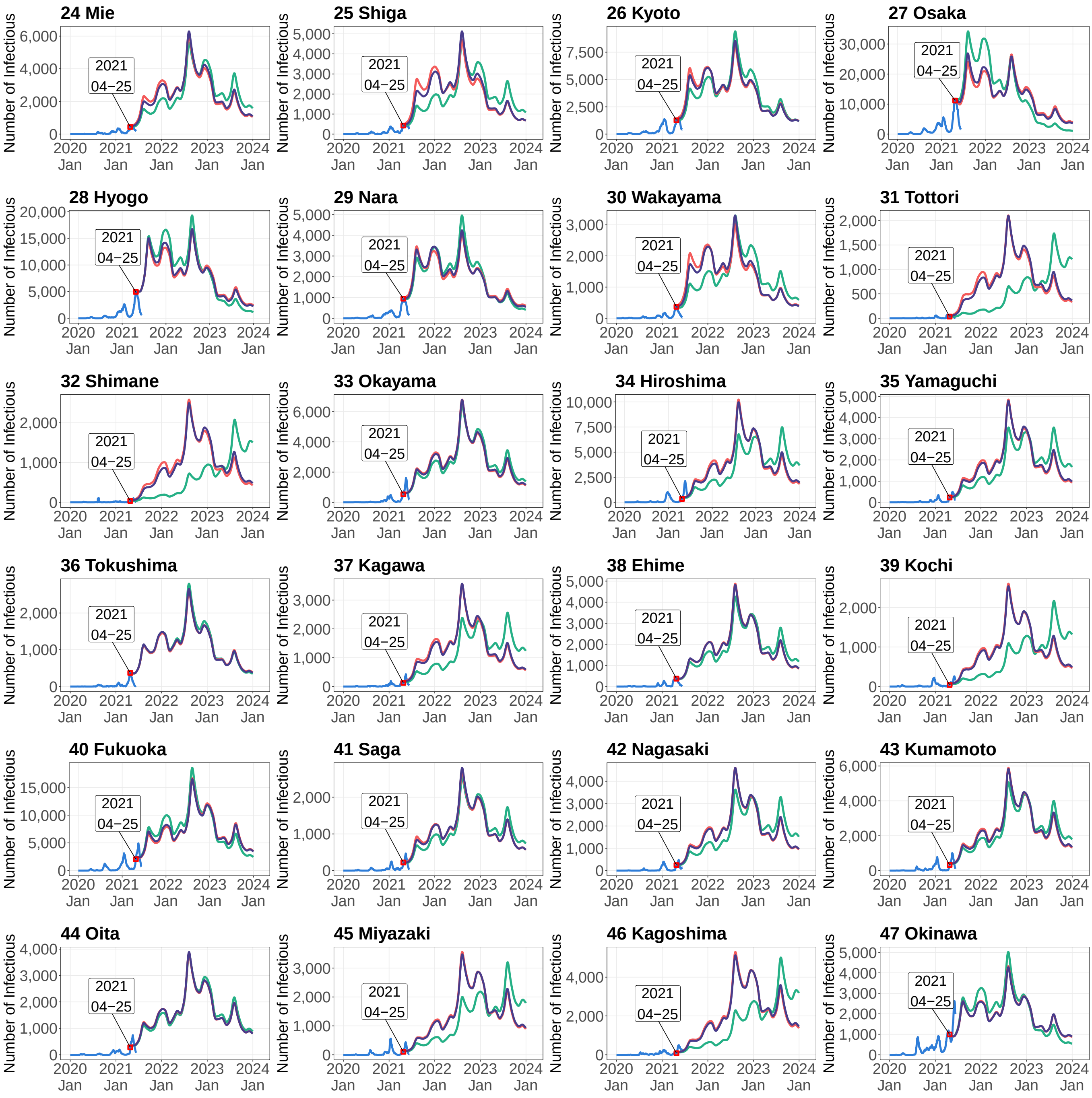

Figure S6. Simulated numbers of infectious people by prefecture in case 3 (*continued*).

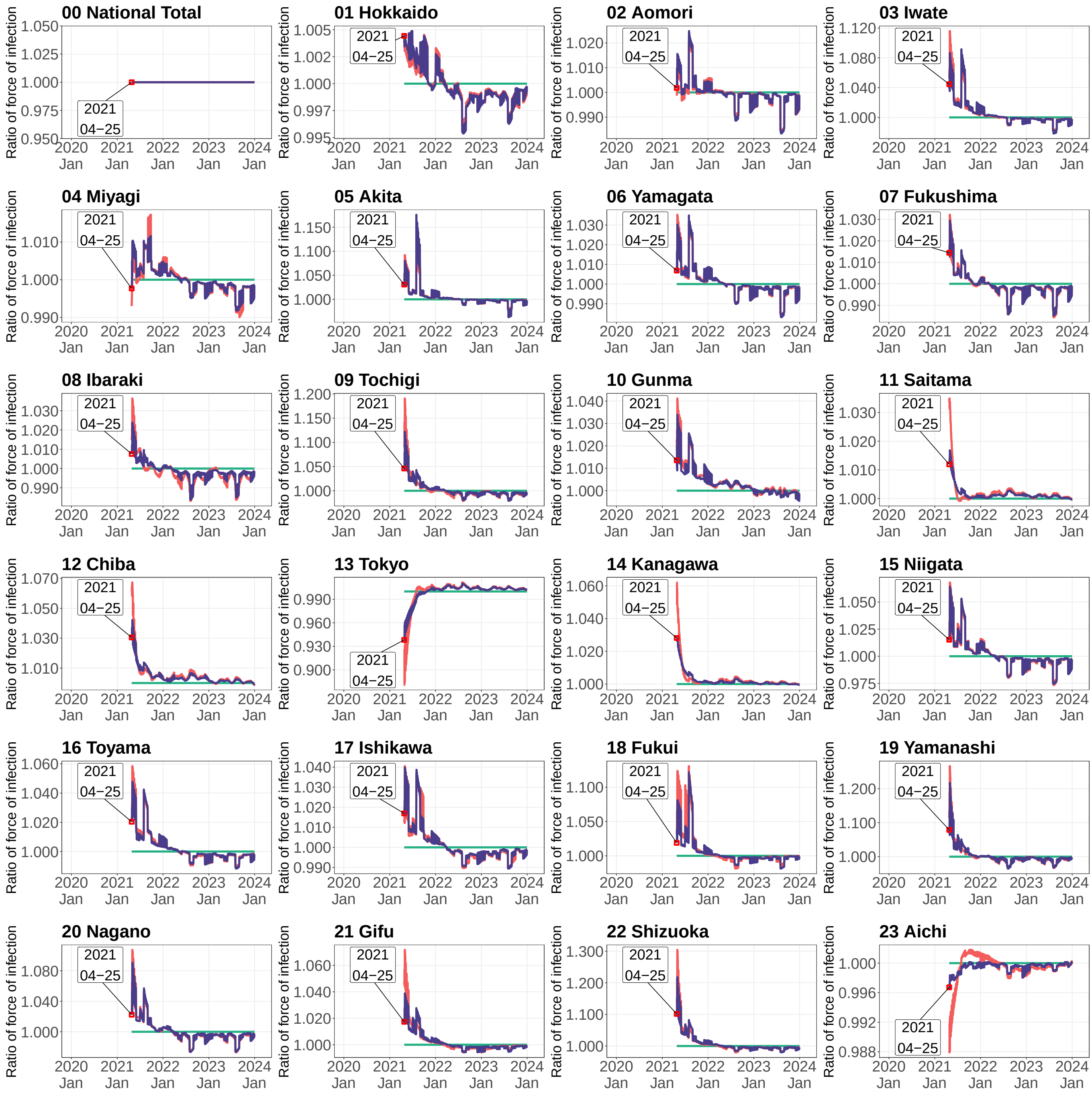

Figure S7. Ratio of daytime and nighttime force of infection by prefecture in case 3. Shown are the ratios of daytime and nighttime force of infection in the spatial SEIR model without interregional mobility (green lines) and those with interregional mobility. The interregional mobility pattern is based on locations where people are located at 2 pm and 8 pm (red and purple lines, respectively).

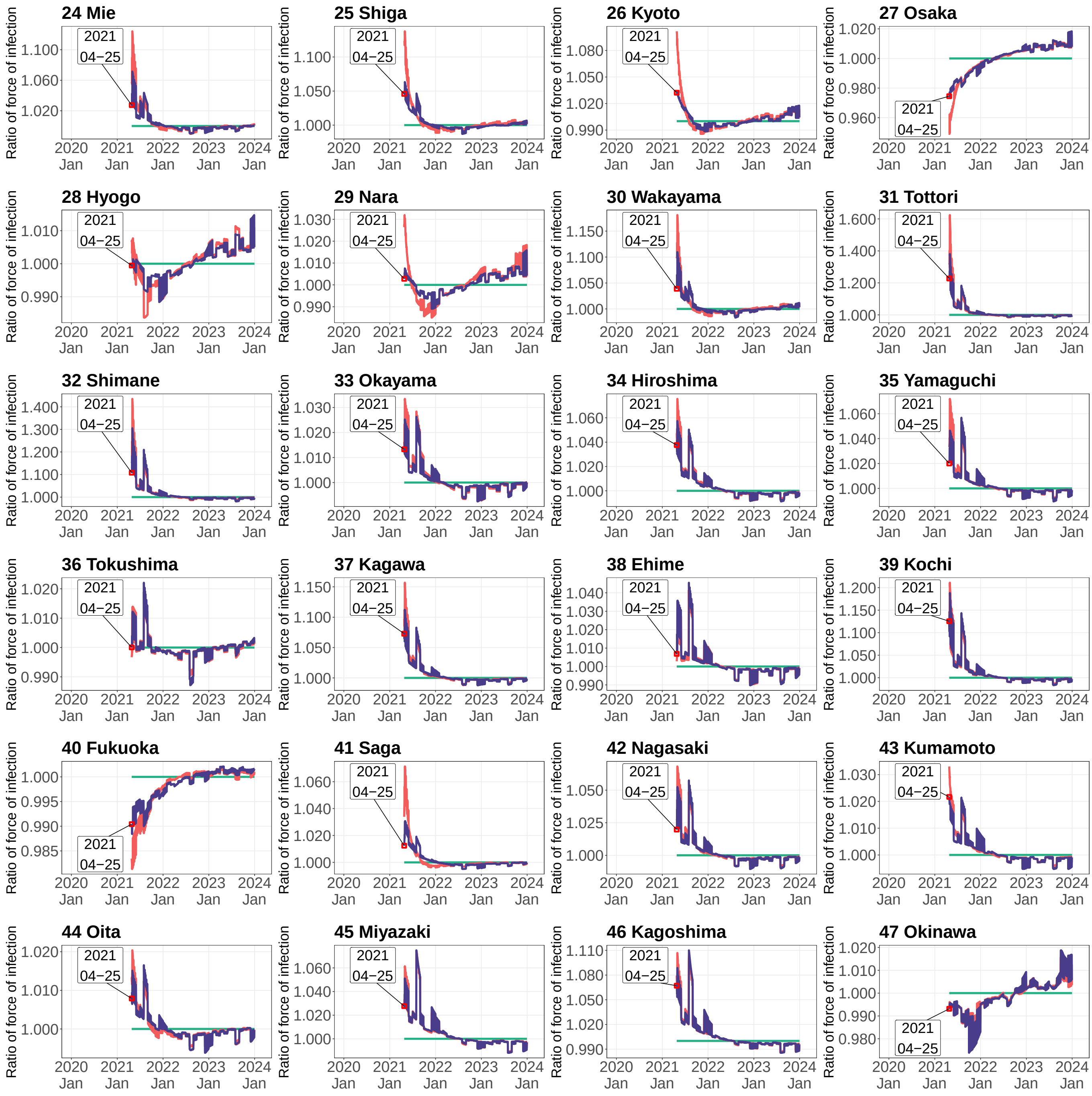

Figure S7. Ratio of daytime and nighttime force of infection by prefecture in case 3 (*continued*).

Appendix S6 Case 4: Interregional mobility restriction only for infectious people

Figures S8 and S9 show the simulation results of the extended spatial SEIR model with the interregional mobility in a situation where the interregional mobility only for infectious people was restricted to remain in their residential prefectures and susceptible, exposed (infected but not yet infectious), and recovered individuals commute or travel across prefectures. In other words, the OD matrix for the infectious people becomes a diagonal matrix, whereas the OD matrix for susceptible, exposed, and recovered people is taken from the mobility data. Case 4 was discussed in the main text.

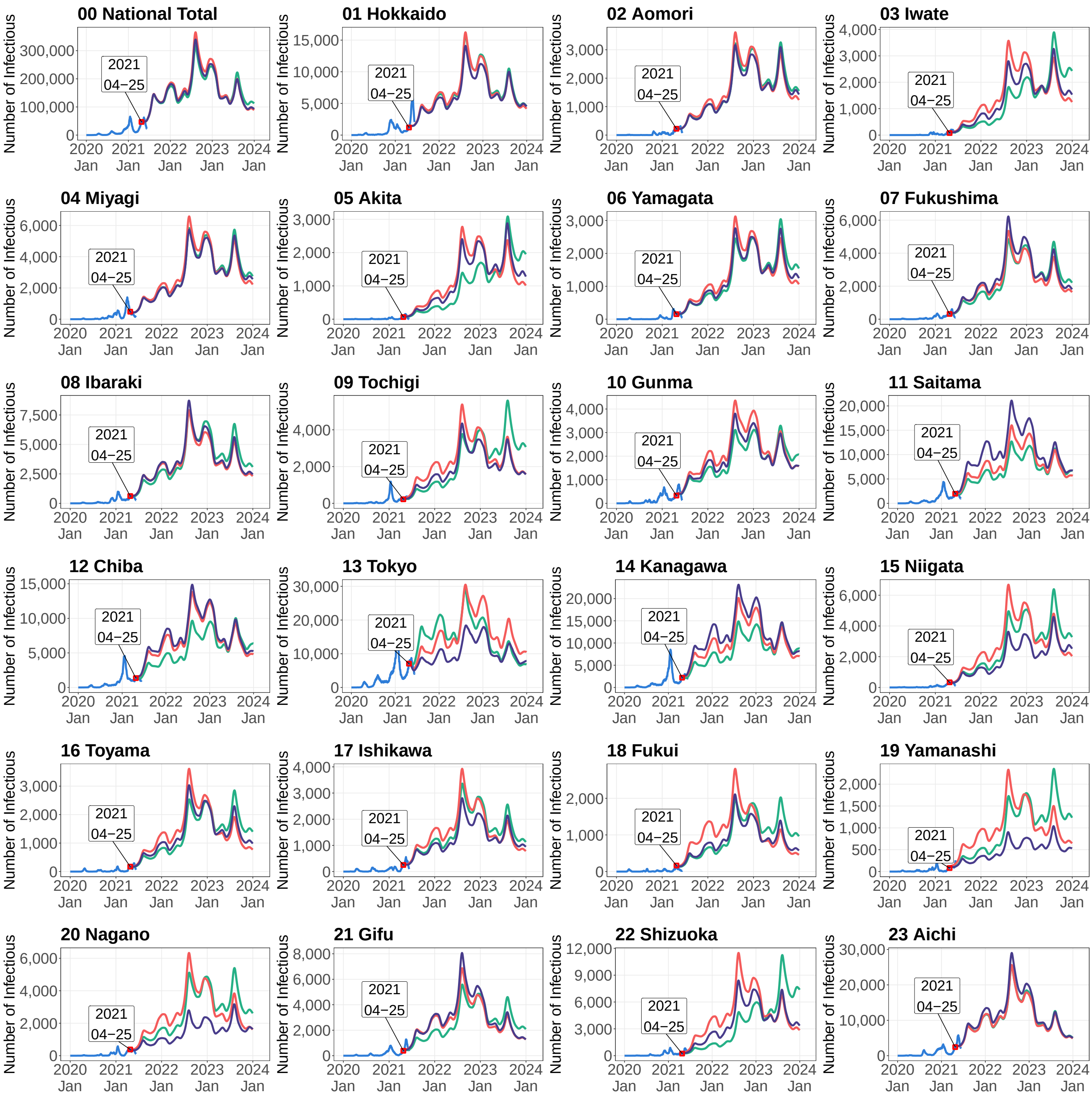

Figure S8. Simulated numbers of infectious people by prefecture in case 4. Shown are the observed numbers of infectious people (blue lines), the numbers of infectious people simulated by the spatial SEIR model without interregional mobility (green lines), and those simulated by the spatial SEIR model with interregional mobility. The interregional mobility of only infectious people is restricted (purple lines). Free mobility across prefectures is allowed for all individuals (red lines).

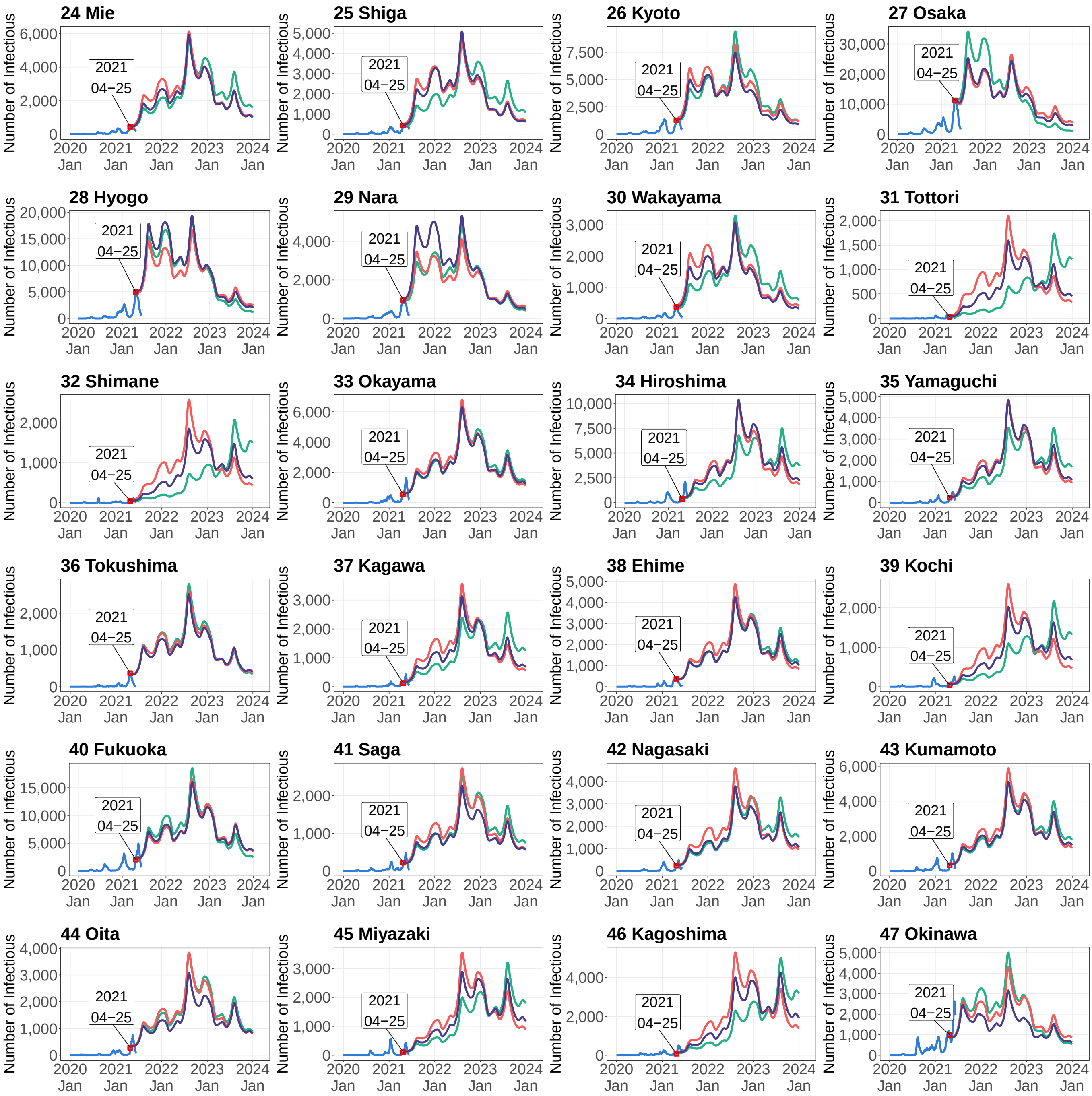

Figure S8. Simulated numbers of infectious people by prefecture in case 4 (*continued*).

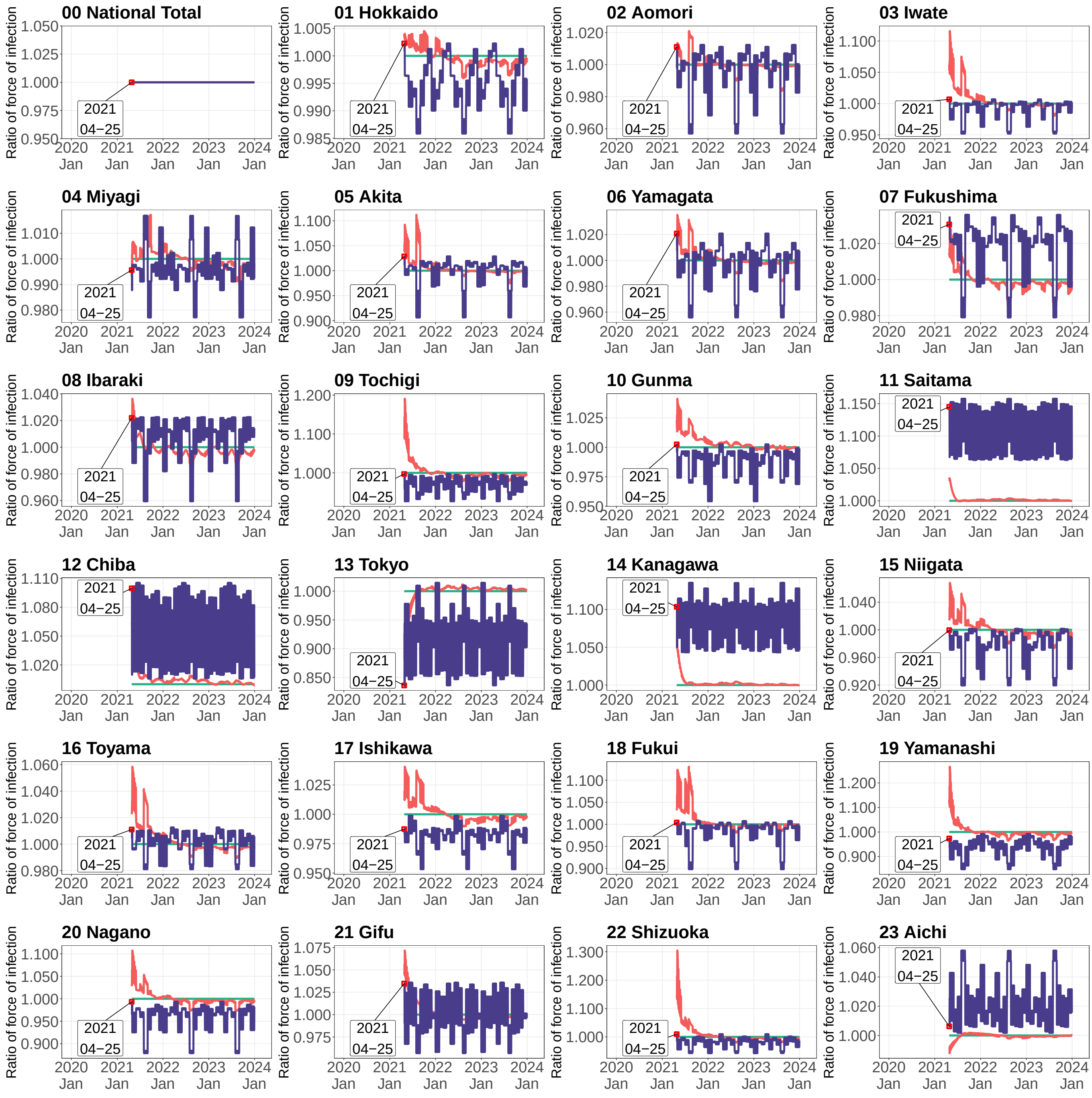

Figure S9. Ratio of daytime and nighttime force of infection by prefecture in case 4. Shown are the ratios of daytime and nighttime force of infection in the spatial SEIR model without interregional mobility (green lines) and those with interregional mobility. The interregional mobility of only infectious people is restricted (purple lines). Free mobility across prefectures is allowed for all individuals (red lines).

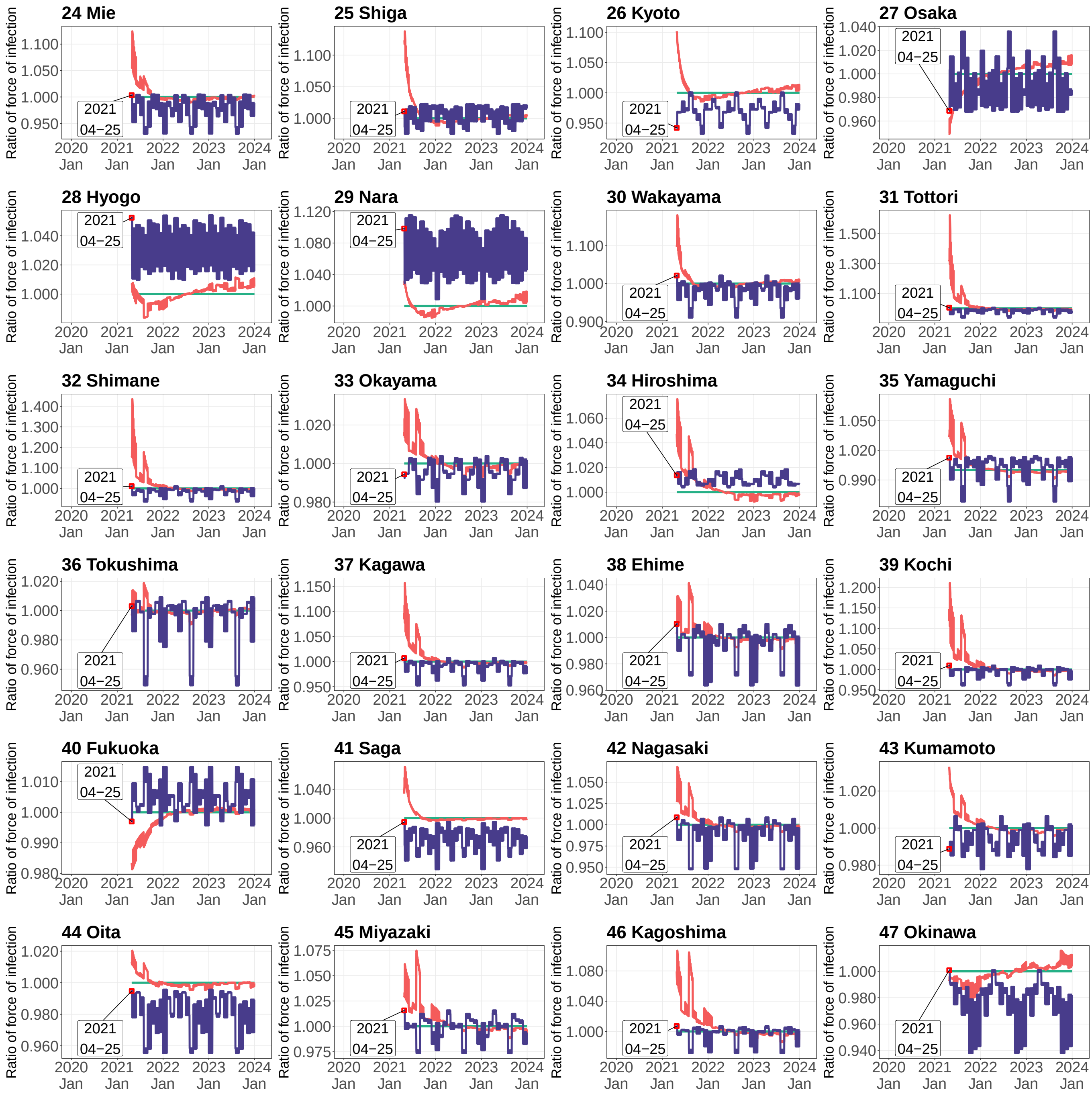

Figure S9. Ratio of daytime and nighttime force of infection by prefecture in case 4 (*continued*).

Appendix S7 Case 5: Interregional mobility restriction only for the Greater Tokyo area

Figures S10 and S11 show the simulation results when the interregional mobility is restricted for the Greater Tokyo area (Saitama, Chiba, Tokyo, and Kanagawa). It is assumed that residents in the Greater Tokyo area are restricted to remain in each prefecture. However, residents in other prefectures are allowed to commute and travel across prefectures, except the Greater Tokyo area.

The simulation results showed that interregional mobility restrictions in the Greater Tokyo area help mitigate the spread of infection in neighboring prefectures, such as Ibaraki, Tochigi, Gunma, Yamanashi, and Shizuoka, because those who reside in these prefectures are exposed to an infection risk in Greater Tokyo area and residents in the Greater Tokyo area tend to visit these prefectures on holidays and weekends. Prefectures in the Tohoku region, such as Aomori, Iwate, Miyagi, Akita, Yamagata, and Fukushima, also show slight reduction by restricting the interregional mobility from and to Greater Tokyo area. However, the interregional mobility from and to the Greater Tokyo area has limited impacts on infection spread in other distant prefectures.

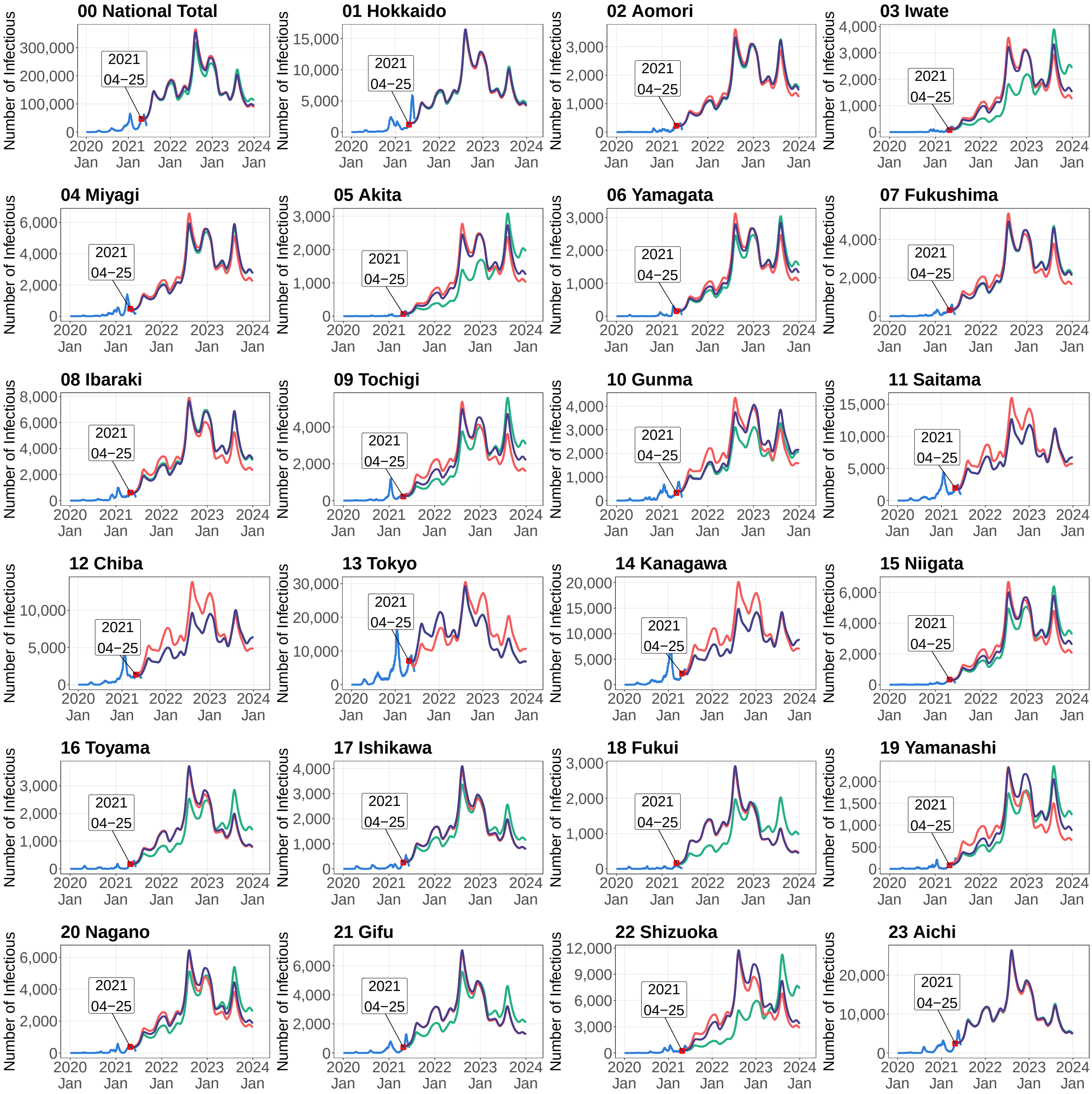

Figure S10. Simulated numbers of infectious people by prefecture in case 5. Shown are the observed numbers of infectious people (blue lines), the numbers of infectious people simulated by the spatial SEIR model without interregional mobility (green lines), and those simulated by the spatial SEIR model with interregional mobility. Residents in the Greater Tokyo area are restricted to remain in each prefecture, and residents in other prefectures are allowed to commute and travel across prefectures, except for the Greater Tokyo area (purple lines). Free mobility across prefectures is allowed for all individuals (red lines).

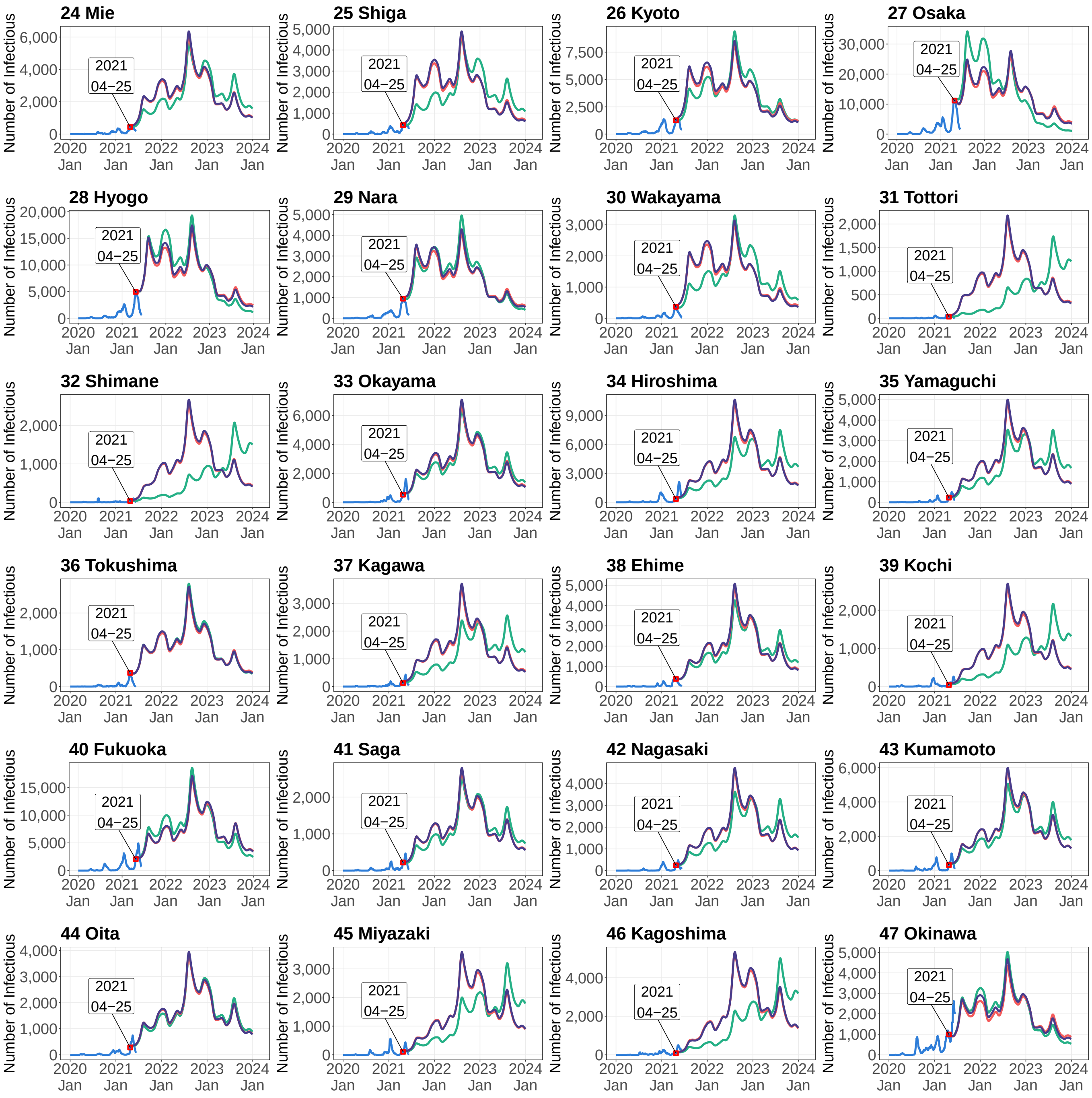

Figure S10. Simulated numbers of infectious people by prefecture in case 5 (*continued*).

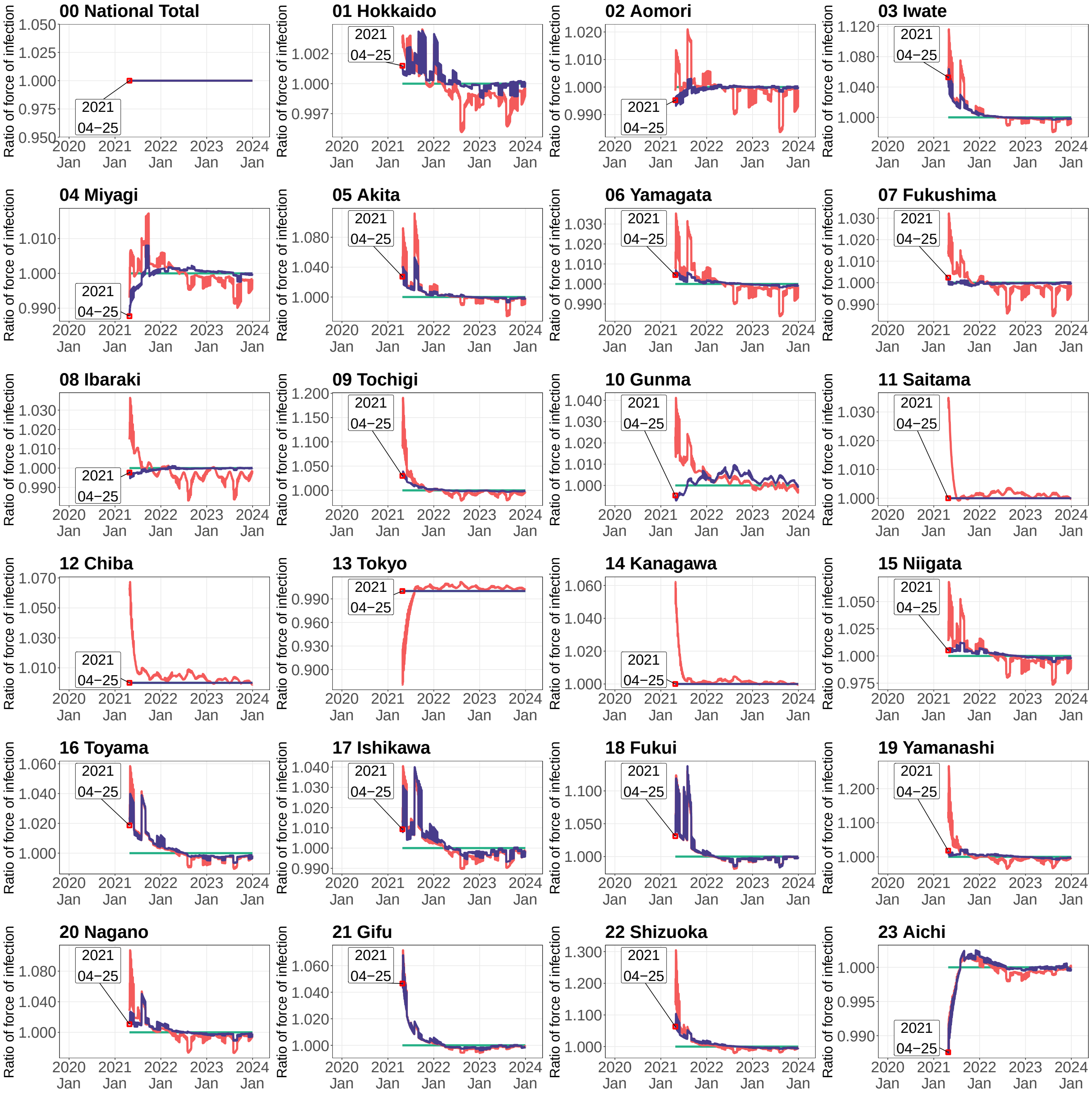

Figure S11. Ratio of daytime and nighttime force of infection by prefecture in case 5. Shown are the ratios of daytime and nighttime force of infection in the spatial SEIR model without interregional mobility (green lines) and those with interregional mobility. Residents in the Greater Tokyo area are restricted to remain in each prefecture, and residents in other prefectures are allowed to commute and travel across prefectures, except for the Greater Tokyo area (purple lines). Free mobility across prefectures is allowed for all individuals (red lines).

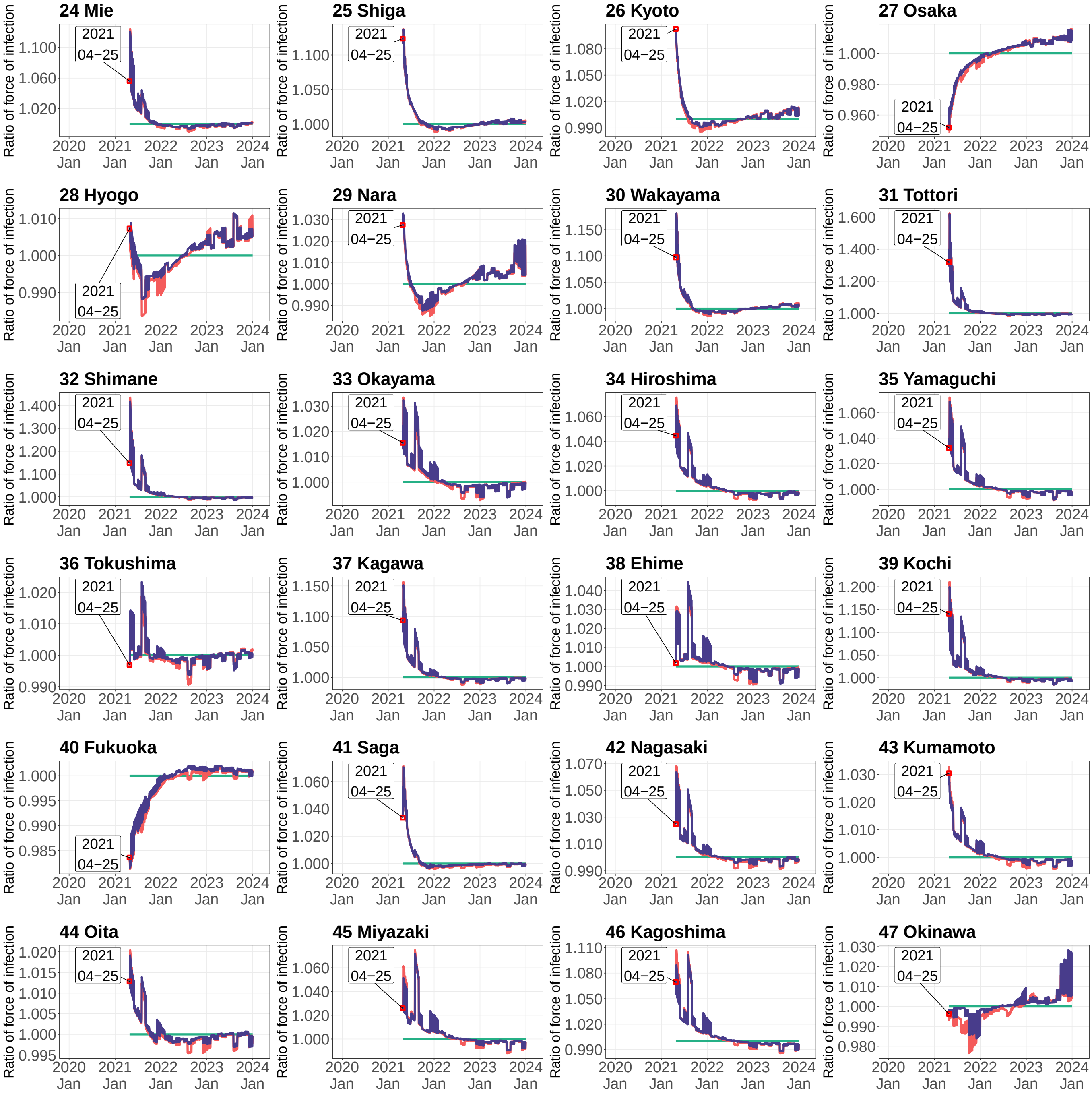

Figure S11. Ratio of daytime and nighttime force of infection by prefecture in case 5 (*continued*).

Appendix S8 Case 6: Interregional mobility restriction only for the Greater Osaka area

Figures S12 and S13 show the simulation results when the interregional mobility is restricted for the Greater Osaka area (Kyoto, Osaka, and Hyogo). It is assumed that residents in the Greater Osaka area are restricted to remain in each prefecture. However, residents in other prefectures are allowed to commute and travel across prefectures, except for the Greater Osaka area.

The simulation results show that the restriction of interregional mobility in the Greater Osaka area mitigates the infection spread to geographically wide prefectures, such as Mie, Shiga, Nara, Wakayama, Tottori, Shimane, Okayama, Hiroshima, Tokushima, Kagawa, Ehime, and Kochi, because these prefectures are connected to the Greater Osaka area. Restricting interregional mobility for the Greater Osaka area prevents the spatial spread of infection to prefectures in the Hokuriku region, such as Toyama, Ishikawa, and Fukui, because the interregional mobility connects these prefectures and the Greater Osaka area. The interregional mobility from/to Greater Osaka area has limited impacts on the spread of infection to other distant prefectures.

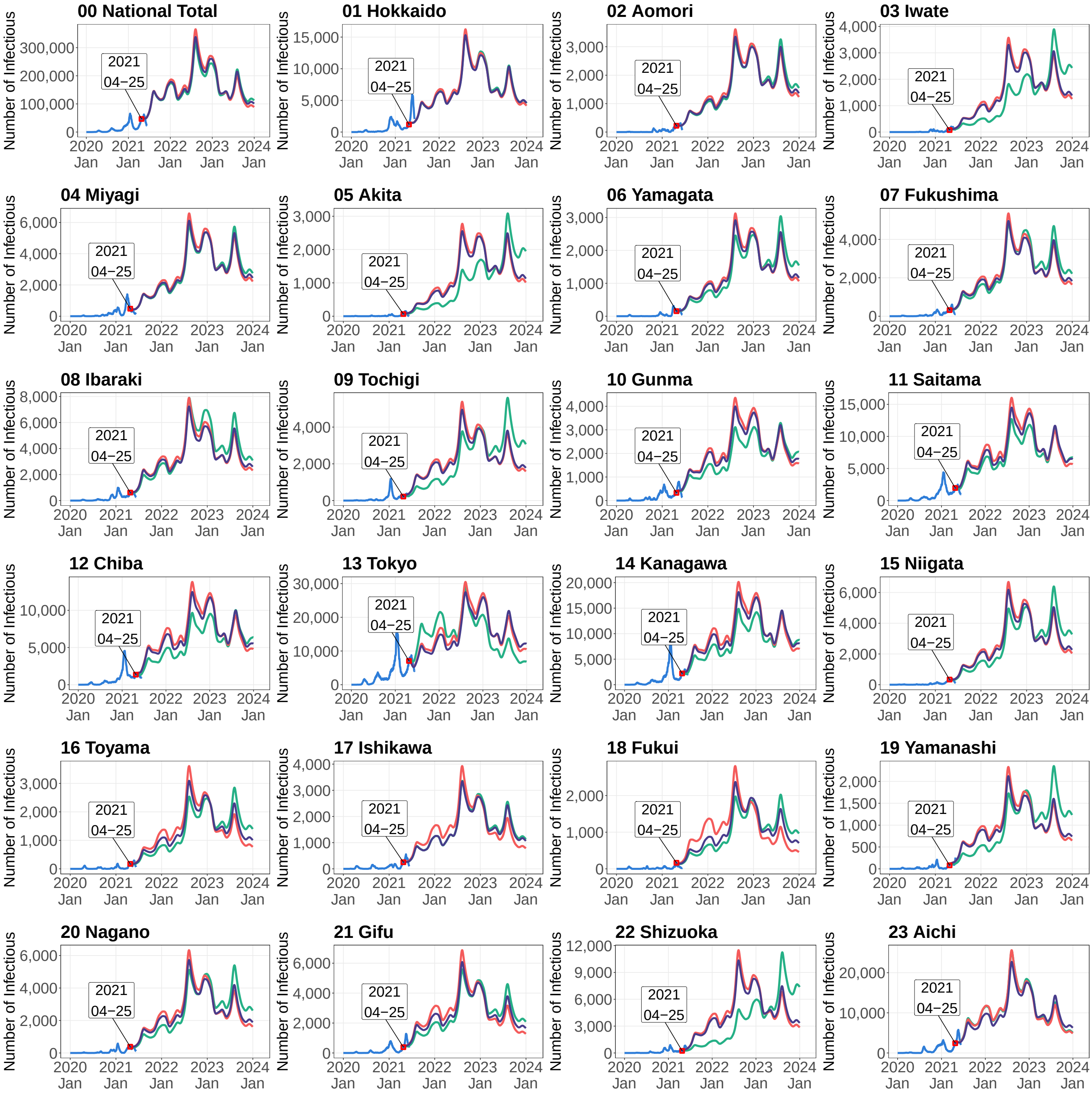

Figure S12. Simulated numbers of infectious people by prefecture in case 6. Shown are the observed numbers of infectious people (blue lines), the numbers of infectious people simulated by the spatial SEIR model without interregional mobility (green lines), and those simulated by the spatial SEIR model with interregional mobility. Residents in the Greater Osaka area are restricted to remain in each prefecture, and residents in other prefectures are allowed to commute and travel across prefectures, except for the Greater Osaka area (purple lines). Free mobility across prefectures is allowed for all individuals (red lines).

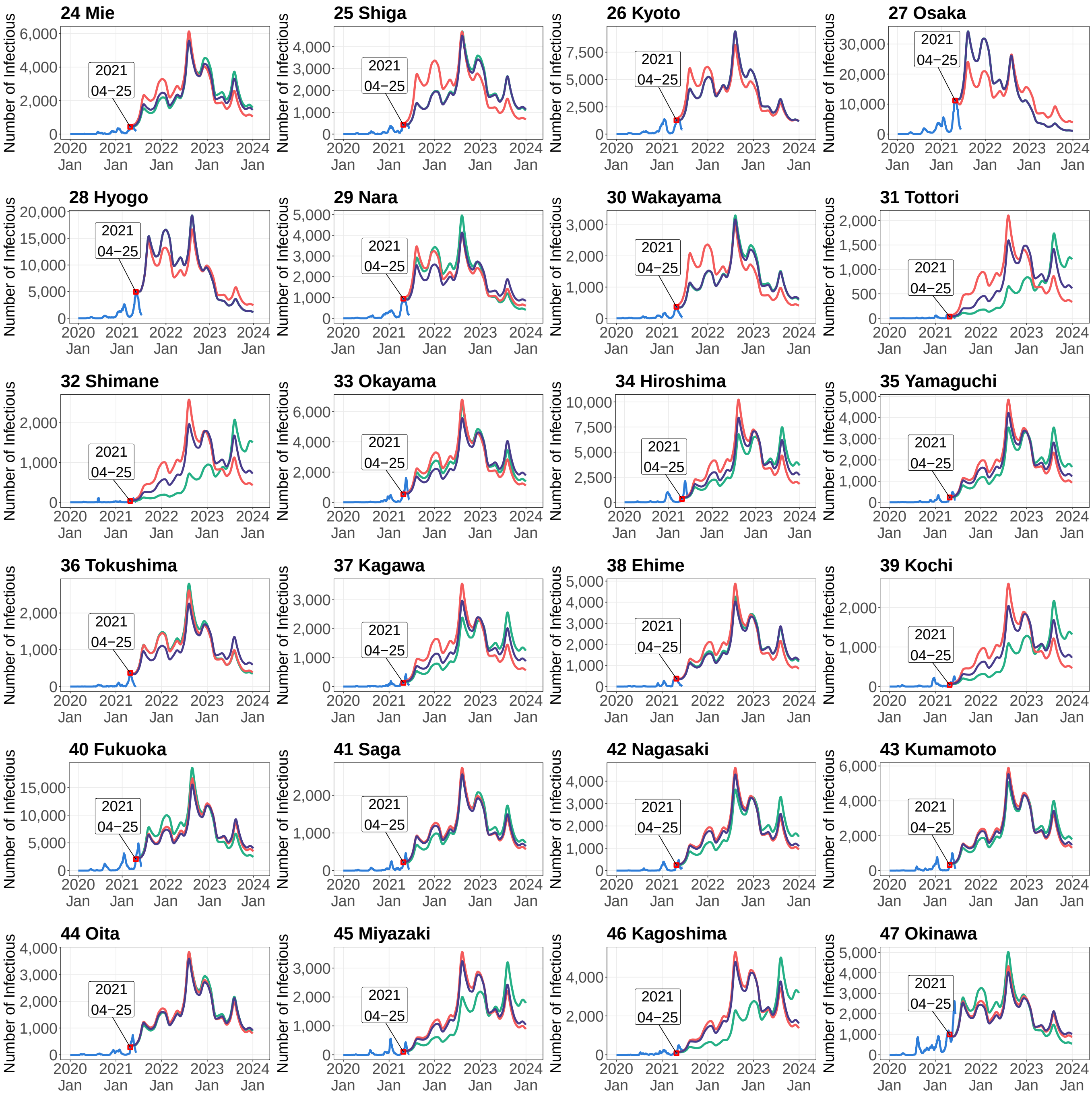

Figure S12. Simulated numbers of infectious people by prefecture in case 6 (*continued*).

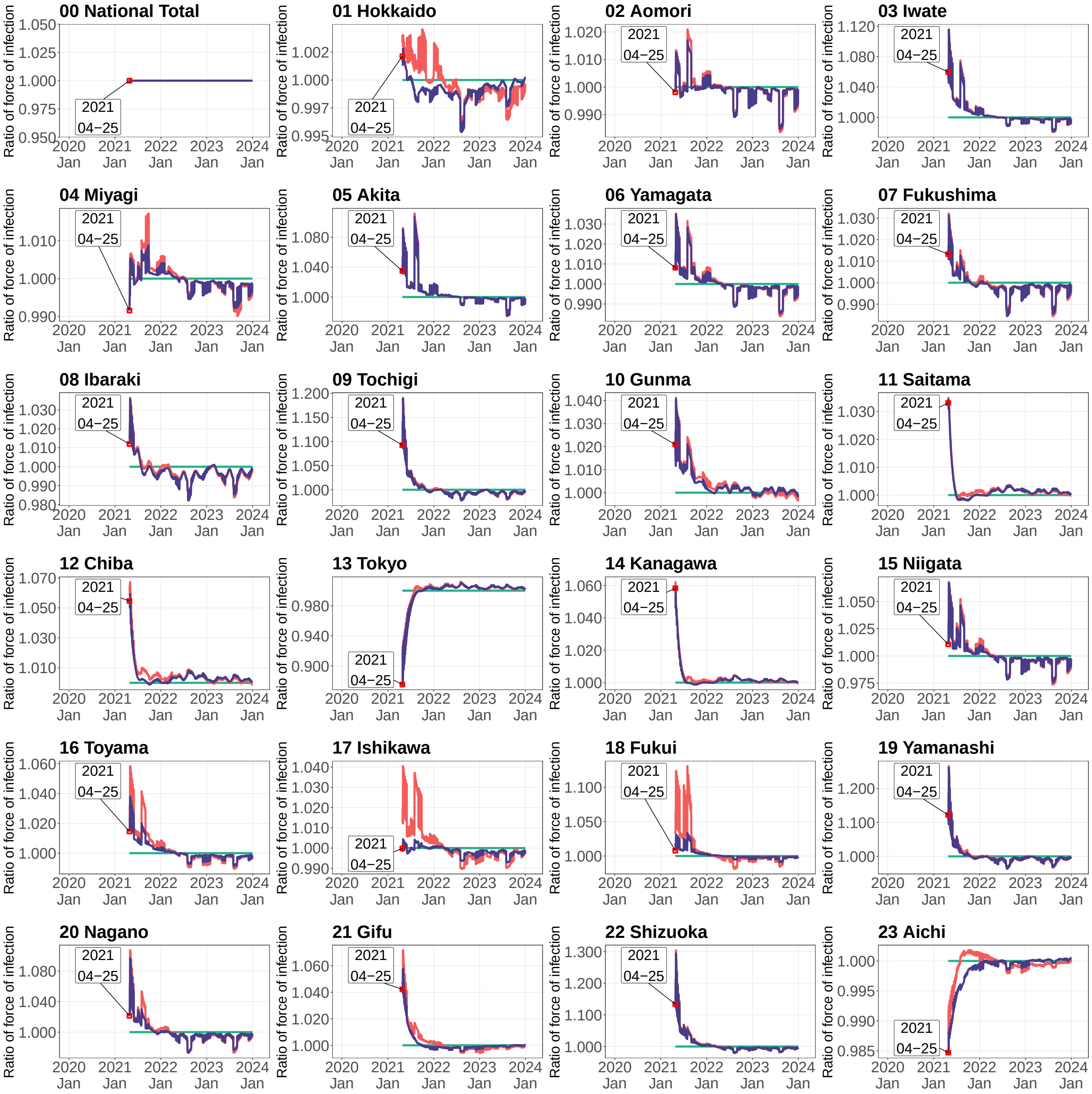

Figure S13. Ratio of daytime and nighttime force of infection by prefecture in case 6. Shown are the ratios of daytime and nighttime force of infection in the spatial SEIR model without interregional mobility (green lines) and those with interregional mobility. Residents in the Greater Osaka area are restricted to remain in each prefecture, and residents in other prefectures are allowed to commute and travel across prefectures, except for the Greater Osaka area (purple lines). Free mobility across prefectures is allowed for all individuals (red lines).

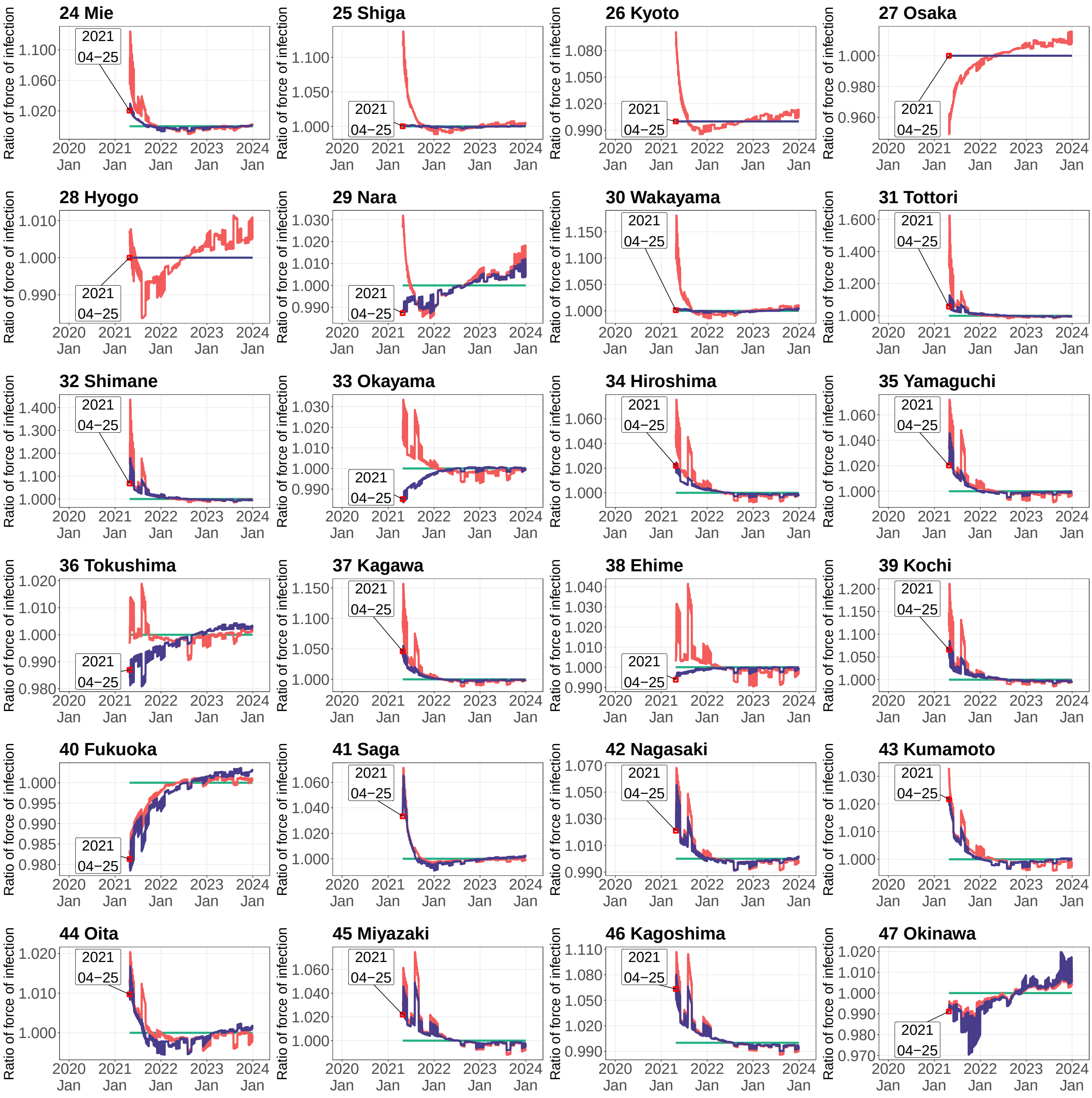

Figure S13. Ratio of daytime and nighttime force of infection by prefecture in case 6 (*continued*).

Appendix S9 Case 7: Interregional mobility restriction only for Tokyo and Osaka

Figures S14 and S15 show the simulation results where the interregional mobility of residents in Tokyo and Osaka is restricted, and residents from other prefectures are allowed to stay in Tokyo and Osaka in the daytime.

The simulation results show that restricting the outflux of individuals from Tokyo and Osaka reduces the infection risk in neighboring prefectures slightly (Saitama, Chiba, Kanagawa, Kyoto, Nara, Wakayama, Tokushima, and Ehime) because the ratio of daytime and nighttime force of infection approaches one. However, the quantitative effects of restricting the outflux of individuals from Tokyo and Osaka to prevent the spatial spread of infection are small, suggesting that the interregional mobility of susceptible individuals causes the influx of SARS-CoV-2 in the neighboring prefectures, resulting in the spatial spread of COVID-19 infections across the country.

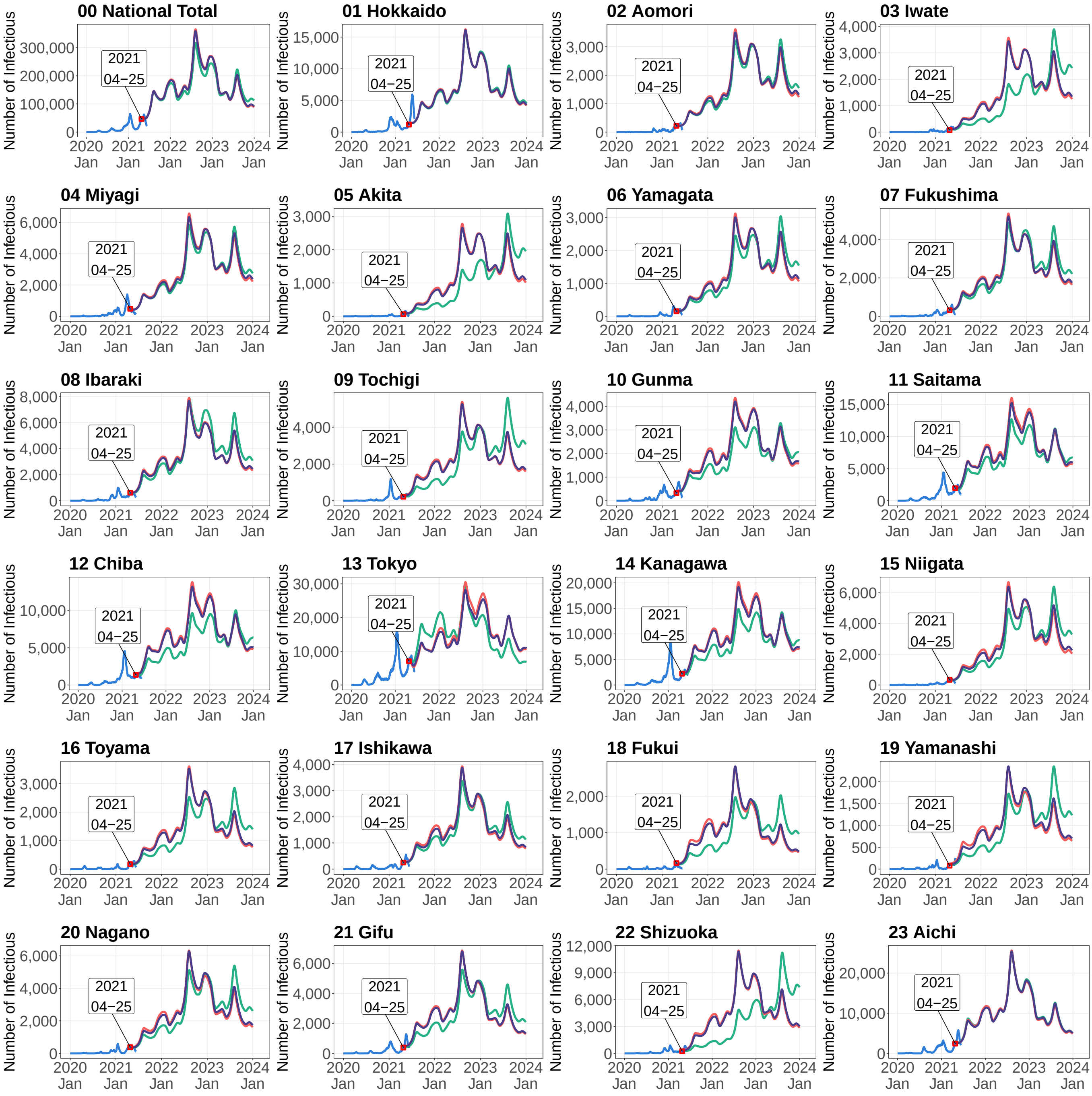

Figure S14. Simulated numbers of infectious people by prefecture in case 7. Shown are the observed numbers of infectious people (blue lines), the numbers of infectious people simulated by the spatial SEIR model without interregional mobility (green lines), and those simulated by the spatial SEIR model with interregional mobility. The interregional mobility of residents in Tokyo and Osaka is restricted and residents in other prefectures are allowed to stay in Tokyo and Osaka in the daytime (purple lines). Free mobility across prefectures is allowed for all individuals (red lines).

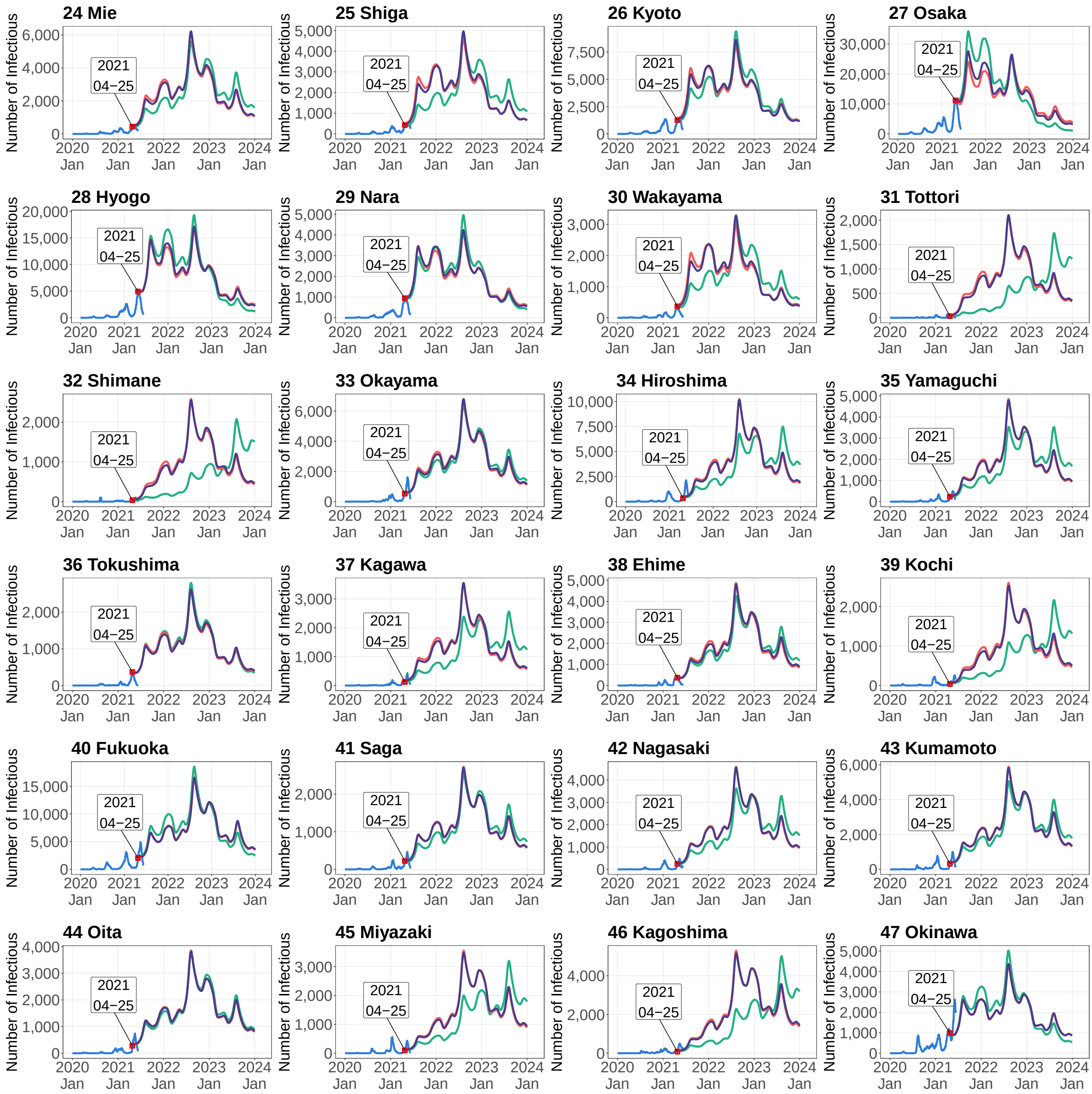

Figure S14. Simulated numbers of infectious people by prefecture in case 7 (*continued*).

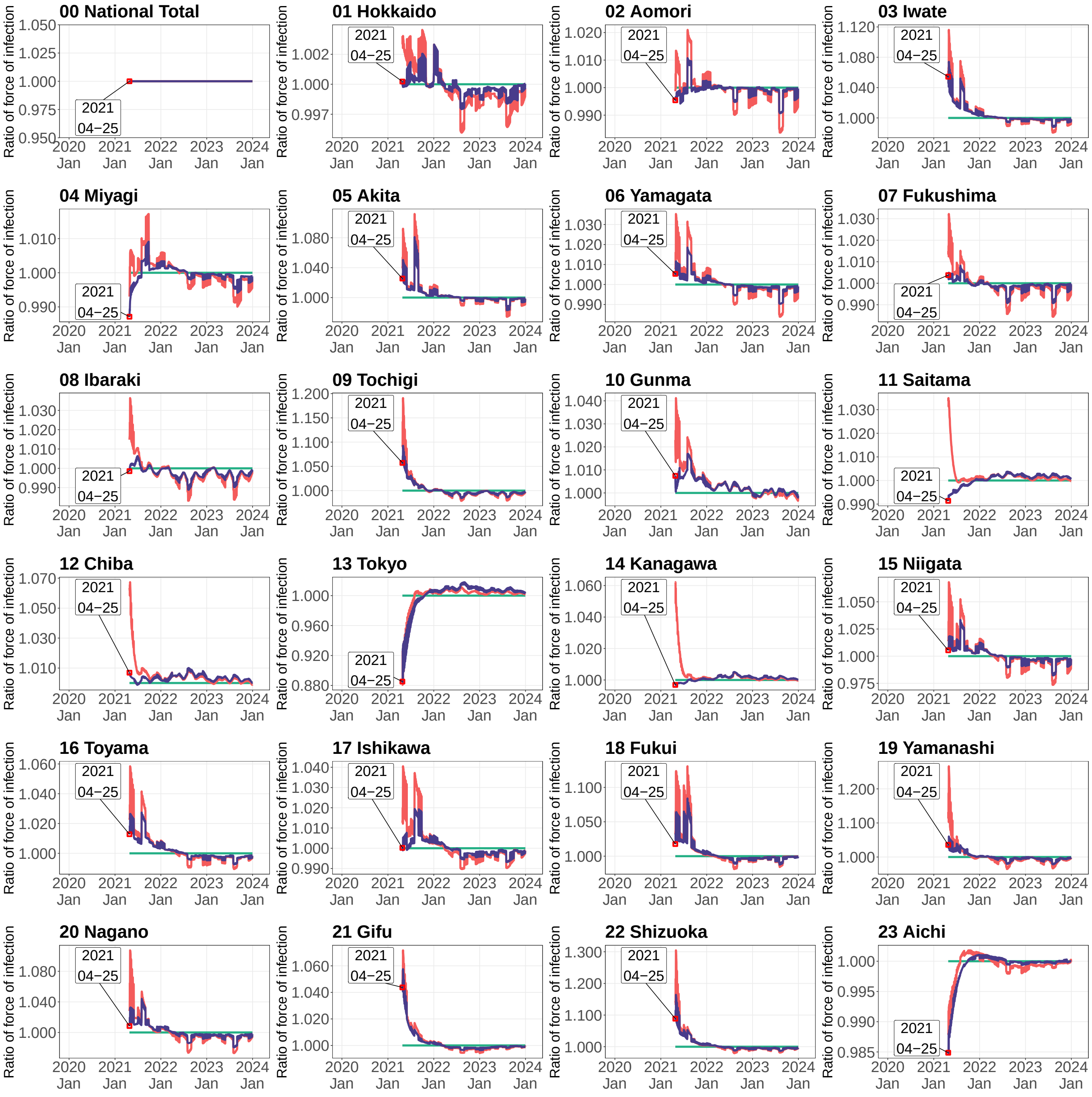

Figure S15. Ratio of daytime and nighttime force of infection by prefecture in case 7. Shown are the ratios of daytime and nighttime force of infection in the spatial SEIR model without interregional mobility (green lines) and those with interregional mobility. The interregional mobility of residents in Tokyo and Osaka is restricted and residents in other prefectures are allowed to stay in Tokyo and Osaka in the daytime (purple lines). Free mobility across prefectures is allowed for all individuals (red lines).

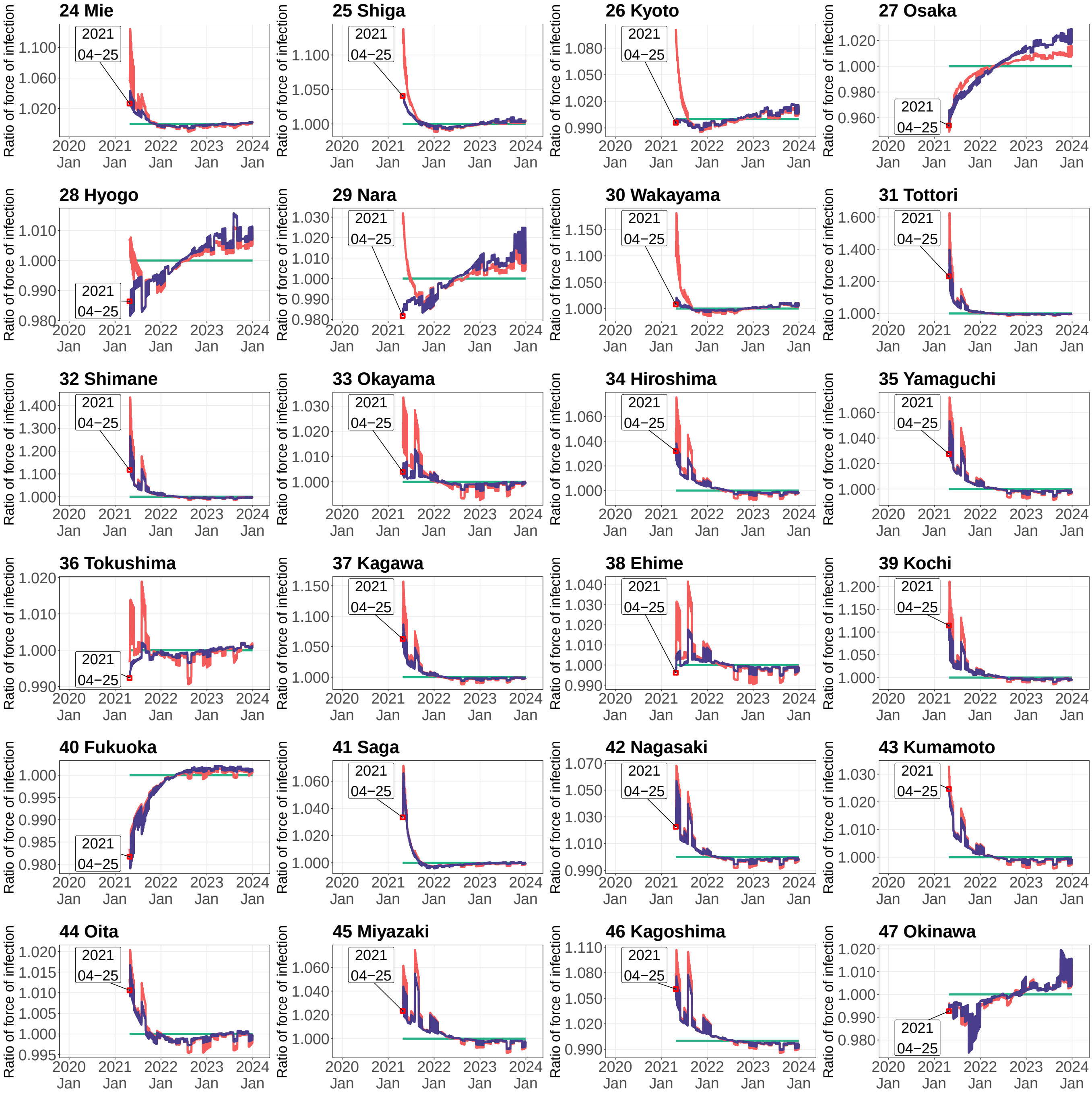

Figure S15. Ratio of daytime and nighttime force of infection by prefecture in case 7 (*continued*).

References

1. Control Office for Novel Coronavirus Disease of Japan. COVID-19 information and resources. `https://corona.go.jp/emergency/#kinkyu` (accessed on 27 July 2021) (2021).

2. Haug, N. et al. Ranking the effectiveness of worldwide COVID-19 government interventions. *Nat. Hum. Behav.* **4**, 1303–1312 (2020).

3. Garchitorena, A. et al. Integrated Packages of Non-Pharmaceutical Interventions Increased Public Health Response Efficiency Against COVID-19 During the First European Wave: Evidence from 32 European Countries. *SSRN Electron. J.* (2020) doi:10.2139/ssrn.3732140.

4. Aravindakshan, A., Boehnke, J., Gholami, E. & Nayak, A. Preparing for a future COVID-19 wave: insights and limitations from a data-driven evaluation of non-pharmaceutical interventions in Germany. *Sci. Rep.* **10**, (2020).
